# Clonal expansion across the seas as seen through CPLP-TB database: A joint effort in cataloguing *Mycobacterium tuberculosis* genetic diversity in Portuguese-speaking countries

**DOI:** 10.1016/j.meegid.2018.03.011

**Published:** 2019-08

**Authors:** João Perdigão, Carla Silva, Jaciara Diniz, Catarina Pereira, Diana Machado, Jorge Ramos, Hugo Silva, Fernanda Abilleira, Clarice Brum, Ana J. Reis, Maíra Macedo, João L. Scaini, Ana B. Silva, Leonardo Esteves, Rita Macedo, Fernando Maltez, Sofia Clemente, Elizabeth Coelho, Sofia Viegas, Paulo Rabna, Amabélia Rodrigues, Nuno Taveira, Luísa Jordao, Afrânio Kritski, José R. Lapa e Silva, Igor Mokrousov, David Couvin, Nalin Rastogi, Isabel Couto, Arnab Pain, Ruth McNerney, Taane G. Clark, Andrea von Groll, Elis R. Dalla-Costa, Maria Lúcia Rossetti, Pedro E.A. Silva, Miguel Viveiros, Isabel Portugal

**Affiliations:** a iMed.ULisboa – Instituto de Investigação do Medicamento, Faculdade de Farmácia, Universidade de Lisboa, Lisboa, Portugal; b Núcleo de Pesquisa em Microbiologia Médica, Faculdade de Medicina, Universidade Federal do Rio Grande, Rio Grande, Rio Grande do Sul, Brazil; c Unidade de Microbiologia Médica, Global Health and Tropical Medicine, GHTM, Instituto de Higiene e Medicina Tropical, IHMT, Universidade Nova de Lisboa, UNL, Lisboa, Portugal; d Centro de Desenvolvimento Científico e Tecnológico (CDCT), Porto Alegre, Brazil; e Departamento de Doenças Infecciosas, Instituto Nacional de Saúde Dr. Ricardo Jorge, Lisboa, Portugal; f Serviço de Doenças Infecciosas, Hospital de Curry Cabral, Lisboa, Portugal; g Hospital da Divina Providência, Serviço de Doenças Infecciosas, Luanda, Angola; h Programa Nacional de Controlo da Tuberculose, Ministério da Saúde de Moçambique, Mozambique; i Instituto Nacional de Saúde, Ministério da Saúde de Moçambique, Mozambique; j Instituto Nacional de Saúde Pública, Projecto de Saúde de Bandim (INASA/PSB), Bissau, Guinea-Bissau; k Centro de Investigação Interdisciplinar Egas Moniz, Instituto Superior de Ciências da Saúde Egas Moniz, Monte de Caparica, Portugal; l Academic Tuberculosis Program, School of Medicine, Federal University of Rio de Janeiro, Rio de Janeiro, Brazil; m Thoracic Diseases Institute, Federal University of Rio de Janeiro, Rio de Janeiro, Brazil; n Laboratory of Molecular Epidemiology and Evolutionary Genetics (former Laboratory of Molecular Microbiology), St. Petersburg Pasteur Institute, St. Petersburg, Russia; o WHO Supranational TB Reference Laboratory, Tuberculosis and Mycobacteria Unit, Institut Pasteur de la Guadeloupe, Morne Jolivière Abymes, Guadeloupe, France; p Pathogen Genomics Laboratory, BESE Division, King Abdullah University of Science and Technology (KAUST), Thuwal, Saudi Arabia; q Lung Infection and Immunity Unit, UCT Lung Institute, University of Cape Town, Groote Schuur Hospital, Observatory, 7925, Cape Town, South Africa; r London School of Hygiene & Tropical Medicine, Keppel Street, London WC1E 7HT, United Kingdom; s Universidade Luterana do Brasil (ULBRA/RS), Porto Alegre, Brazil

**Keywords:** MIRU-VNTR, Spoligotyping, Tuberculosis, Drug resistance, Migration, Mycobacteria, LAM

## Abstract

Tuberculosis (TB) remains a major health problem within the Community of Portuguese Language Speaking Countries (CPLP). Despite the marked variation in TB incidence across its member-states and continued human migratory flux between countries, a considerable gap in the knowledge on the *Mycobacterium tuberculosis* population structure and strain circulation between the countries still exists. To address this, we have assembled and analysed the largest CPLP *M. tuberculosis* molecular and drug susceptibility dataset, comprised by a total of 1447 clinical isolates, including 423 multidrug-resistant isolates, from five CPLP countries. The data herein presented reinforces Latin American and Mediterranean (LAM) strains as the hallmark of *M. tuberculosis* populational structure in the CPLP coupled with country-specific differential prevalence of minor clades. Moreover, using high-resolution typing by 24-loci MIRU-VNTR, six cross-border genetic clusters were detected, thus supporting recent clonal expansion across the Lusophone space.

To make this data available to the scientific community and public health authorities we developed CPLP-TB (available at http://cplp-tb.ff.ulisboa.pt), an online database coupled with web-based tools for exploratory data analysis. As a public health tool, it is expected to contribute to improved knowledge on the *M. tuberculosis* population structure and strain circulation within the CPLP, thus supporting the risk assessment of strain-specific trends.

## Highlights

• The Community of Portuguese Speaking Countries (CPLP) occupies a vast geographical area.
• Three CPLP countries are shortlisted in the WHO's list of Top 30 high-burden countries.
• Common *Mycobacterium tuberculosis* population structure denote historical strain flow.
• Cross-border clusters suggest recent intercontinental tuberculosis transmission.
• CPLP-TB: a novel strain database and framework for collaborative studies and strain tracing.

## 1 Introduction

Tuberculosis (TB) is a devastating disease and a major public health issue, particularly among developing countries. The World Health Organization (WHO) declared TB as a global emergency in 1993 (World Health Organization, 1994). In 1997, new TB cases were estimated to ascend to approximately 8 million new cases per year and TB alone was responsible for 1.9 million deaths (Dye et al., 1999). Almost two decades later, the WHO estimates a total of 10.4 million new incident cases and 1.79 million deaths due to TB in 2015 (World Health Organization, 2016). The increasing trend in TB incidence shown by some countries and the fact that some of these had a larger incidence level than that previously recognized, have contributed to the massive number of TB cases we face today (World Health Organization, 2016).

The Community of Portuguese Speaking Countries (CPLP - https://www.cplp.org) comprises of nine member-states sharing a common trait – Lusophony (i.e., Portuguese-spoke language). Together, the member states account for 10,742,000 Km^2^ (7.2%) of the world's land area and contain around 258,466,109 habitants (approx. 3.5% of the human population). These countries accounted for 348,600 (3.4%) of the new TB cases in 2015 and, together, they show an average incidence rate of 87.5 new cases per 100,000 habitants (World Health Organization, 2016). This high incidence rate is not uniform across all member-states, ranging from 23 (Portugal) to 551 (Mozambique) new cases per 100,000 habitants. Noteworthy, three of these countries (Angola, Brazil and Mozambique) are within the WHO's Top 30 High-Burden countries for TB (World Health Organization, 2016).

*Mycobacterium tuberculosis*, the etiological agent of TB, has shown a highly clonal population structure but even with the present state-of-the-art molecular fingerprinting methods or Whole Genome Sequencing, we still miss some of the links in these transmission chains and occasionally we fail to understand how *M. tuberculosis* is transmitted in the community (van Soolingen, 2014; Walker et al., 2013).

Still, the wide application of CRISPR-based spoligotyping, along with other genetic markers such as IS*6110*, IS*1081* and VNTR *loci* for isolate fingerprinting has enabled the delineation of broader but distinct genetic lineages and their phylogeographical association and, lineages such as Latin American and Mediterranean (LAM) or the East African-Indian (EAI) were subsequently assigned characteristic spoligotyping profiles (Sola et al., 2001). However, spoligotyping interrogates specific spacer sequences within the Direct Repeat (DR) *locus* which evolves unidirectionally by independent loss of single spacers or through the deletion of contiguous repeats and spacers (Groenen et al., 1993). This unidirectional and bimodal mode of evolution may be difficult to interpret, model and, foremost, can lead to homoplasy which can encompass a non-negligible effect on strain clustering by spoligotyping alone (Comas et al., 2009; Reyes et al., 2012). In this sense, VNTR-based typing emerged as a genotyping method that is less prone to convergence by relying on the analysis of interspersed multi-allelic *loci* (Supply et al., 2006). Despite this, some of the spoligotyping main lineages or families were later confirmed and defined using new molecular markers such as the SNP barcode proposed by Coll et al. (2014). The prevalence of these lineages and clades at country level has gradually been made available through databases such as SITVITWEB or MIRU-VNTR*plus*, enabling the study of the *M. tuberculosis* population structure across different countries (Couvin and Rastogi, 2015; Demay et al., 2012; Weniger et al., 2010).

In the CPLP countries the body of knowledge available in the literature and databases has revealed that the *M. tuberculosis* populational structure contains a high predominance of LAM strains (Demay et al., 2012; Groenheit et al., 2011; Mokrousov et al., 2016; Perdigao et al., 2017; Saifodine et al., 2014; Viegas et al., 2010). However, an intense flow of people has historically existed between these countries and does still presently occur (http://migrationsmap.net). For this reason, additional tools developed for these specific countries are necessary to rapidly disseminate molecular epidemiological data and enable a higher resolution analysis of TB transmission.

The prevalence of drug resistance is highly variable across different CPLP member states. According to the WHO, rifampicin (RIF) resistant and multidrug resistant (MDR) TB, i.e. resistance to at least isoniazid (INH) and RIF, which is associated with poorer treatment outcome and mortality, may have ascended to 14,292 cases across the CPLP in 2015 (World Health Organization, 2016). Moreover, there is mounting evidence that the distribution of drug resistance is associated with specific phylogenetic clades and that specific within-country settings are associated with higher drug resistant TB rates than those currently estimated (Perdigao et al., 2017; Perdigao et al., 2010). The ability to track clones associated with such phylogenetic clades is therefore of the utmost importance in preventing the emergence of drug-resistant strains, including MDR- and XDR-TB, throughout the different countries.

Here, we combined different datasets from several member-states to provide a new framework for tracing specific *M. tuberculosis* strains across the Lusophone space and developed CPLP-TB, an online database aimed at disseminating molecular epidemiological data.

## 2 Methods

### 2.1 Clinical isolates and datasets

Five distinct datasets (Angola, n = 89; Brazil, n = 964; Guinea-Bissau, n = 13; Mozambique, n = 14; and, Portugal, n = 367) totalling 1447 *M. tuberculosis* clinical isolates (all of which recovered from different patients) were studied:

• Angolan dataset (n = 89): comprised by clinical isolates recovered from sputum samples obtained between March to June 2015 from the Hospital of Divina Providência in Luanda which, serves an estimated population of 990,892 inhabitants. This sample comprises approximately 18% of this hospital's yearly diagnosed cases;
• Brazil dataset (n = 964): this dataset is comprised by isolates from Rio Grande recovered at University Federal do Rio Grande (n = 624, 2006–2016) covering 30% of all TB cases and, 100% of the TB/HIV cases in the city of Rio Grande in this period; and, by MDR-TB/INH-resistant isolates from Porto Alegre and surrounding metropolitan area obtained from the LACEN - Rio Grande do Sul (n = 340, 2010–2014), estimated to cover 50% of such cases in the same period;
• Guinea-Bissau (n = 13): includes all MDR-TB strains collected in Bissau, Guinea-Bissau, at Hospital Raoul Follereau, during the period from May to December 2012;
• Mozambique (n = 14): this dataset includes DNA obtained from isolates recovered at Laboratório Nacional de Referência da Tuberculose from patients diagnosed at Hospital Central de Maputo between January to March 2012;
• Portugal (n = 367): includes isolates recovered from nine distinct laboratories and hospitals across Lisbon Health Region between 1995 and 2016. Most cases (n = 349) were obtained between 2007 and 2015 and include 129 MDR-TB cases that are estimated to correspond to 65% of all the MDR-TB cases reported nationwide for the same period.

The inclusion criterion for this study was the availability of genotypic data: MIRU-VNTR (either 15 or 24 loci) and/or spoligotyping. All methods were performed in accordance with the relevant guidelines and regulations.

### 2.2 Drug susceptibility testing (DST)

DST, when available, was carried out for first-line drugs using the standardized procedure of the BACTEC™ MGIT™ 960 System (Becton Dickinson Diagnostic Systems, Sparks, MD, USA), except for a subset (n = 283/964) of the isolates from Brazil for which phenotypic drug susceptibility was assessed by the resazurin microtiter assay (REMA) (Palomino et al., 2002). Phenotypic data on pyrazinamide (PZA) susceptibility was only available for 90 out of the 713 isolates tested for first-line drug susceptibility in Brazil since PZA susceptibility is not routinely performed in this country. For this same reason, clinical isolates recovered in Portugal before 2000 also lack data on PZA susceptibility.

DST data for second-line drugs (SLDs) was available for 48, 9 and 98 MDR-TB clinical isolates from Brazil, Guinea-Bissau and Portugal, respectively. For this study, only phenotypic susceptibility data for fluoroquinolones (FQ; ofloxacin, ciprofloxacin or moxifloxacin) and second-line injectable drugs (SLIDs; kanamycin, amikacin or capreomycin) were considered.

Clinical isolates susceptible to all first-line drugs tested were classified as pan-susceptible isolates; resistance to at least isoniazid (INH) and rifampicin (RIF) was classified as MDR; any resistance pattern including resistance to one or more antituberculous drugs other than INH and RIF combined were classified as non-MDR; MDR-TB isolates showing cumulative resistance with any FQ and a second-line injectable drug were classified as extensively drug resistant (XDR). MDR-TB isolates lacking data on SLD susceptibility were, nonetheless, classified as MDR-TB.

### 2.3 MIRU-VNTR typing

Fifteen- or 24-loci MIRU-VNTR typing was carried out using the single or multiplex amplification procedure described by Supply et al. (2006). Amplicon size determination was carried out by agarose gel electrophoresis or capillary electrophoresis as described previously (Supply, 2005). Allelic diversity (*h*) for each locus was calculated using the equation *h* = 1 − Σ*x*_*i*_^2^, where *x*_*i*_ is the relative frequency of the *i* th allele (Sun et al., 2004). A MIRU-VNTR dendrogram was constructed using R and ape package for phylogenetic analysis (Paradis et al., 2004). A MIRU-VNTR cluster was defined as a group of more than one isolate sharing indistinguishable MIRU-VNTR profiles and a cross-border cluster defined as a MIRU-VNTR cluster where all clinical isolates do not originate from the same country. Isolates showing two or more loci with dual allele profiles (assessed by dual peak or dual banding upon capillary/gel electrophoresis) were removed from the study.

### 2.4 Spoligotyping

Spoligotyping was performed by a single-tube multiplex PCR amplification of 43 spacer regions of the direct repeat (DR) locus using oligonucleotide primers DRa (5′-Biotin-GGTTTTGGGTCTGACGAC-3′) and DRb (5′-CCGAGAGGGGACGAAAC-3′) and 20 ng of genomic DNA. Amplicons were reverse hybridized on a membrane with amino-linked immobilized probes for each spacer as described previously by Kamerbeek et al. (1997). Detection was performed using the ECL® Chemiluminescence Detection System (GE Healthcare®, Cleveland, OH, USA) as per the manufacturer's instructions.

Spoligotyping profiles were assigned to lineage, clade and shared international type (SIT) using the rules described in SITVITWEB and SITVIT2 international databases (Couvin and Rastogi, 2015; Demay et al., 2012).

SIT assignment was carried out by entering the data in an updated proprietary version of SITVIT (SITVITEXTEND, housed at Institut Pasteur de la Guadeloupe). Minimum Spanning Trees (MST) were drawn using MLVA Compare software (Genoscreen, Lille, France and Ridom Bioinformatics, Münster, Germany).

### 2.5 Database development

CPLP-TB user interface was developed and implemented with Shiny by Rstudio (http://shiny.rstudio.com). Data processing, map and tree plots were implemented using the following R packages: ggplot2, ape, plotGoogleMaps, ggtree and Cairo (Paradis et al., 2004). Interactive choropleth mapping of clinical isolates by country of origin was carried out using Google Maps API. Source and data processing code is available upon request from the corresponding author.

The web application contains a left-sided panel that enables the user to apply filters (e.g. year interval, country of origin, drug resistance, SIT/Clade, MIRU-VNTR cluster, etc) to the data being displayed on a main panel. This main panel is automatically updated according to the filtering criteria set on the left panel, and features different page tabs: a data table that lists all isolates' records deposited in this database along with phenotypic and genotypic data, allowing the user to browse through the different clinical isolates and apply additional filters to the data table; different plots that summarize data contained in the database, e.g., barplots showing the distribution per year, resistance type or SITs/clade; an interactive Google Maps API-based choropleth map showing the distribution of isolates per country; MIRU-VNTR and spoligotyping dendrograms; and, a genome-wide SNP-based phylogenetic tree containing the isolates present in the database that have already been subjected to Whole Genome Sequencing, annotated with the different SNP barcode sub-lineages previously identified by Coll et al. (2014). In its first and current release, CPLP-TB accommodates the datasets described in the present study.

### 2.6 Statistical analysis

All statistical analyses were conducted using the IBM© SPSS© Statistics v.21 (IBM Corporation, Armonk, NY, USA) or R (R Foundation for Statistical Computing, Vienna, Austria).

## 3 Results and discussion

### 3.1 Dataset description: country of origin, isolation years and drug resistance

Through a collaborative effort, the largest of this kind aimed at cataloguing the genetic diversity of *M. tuberculosis* across CPLP countries, this study brought together five distinct datasets comprised by *M. tuberculosis* clinical isolates recovered in five distinct CPLP countries: Angola (n = 89); Brazil (n = 964); Guinea-Bissau (n = 13); Mozambique (n = 14); and, Portugal (n = 367). These clinical isolates comprise a convenience sample collected from several laboratories and hospital units (see Methods for further details) between 1995 and 2016 (Table 1). The distribution per year of these clinical isolates and the number of drug resistant isolates to first-line drugs is summarized in Table 1. Briefly, from a total 1447 clinical isolates included in this study, 1182 (81.7%) were subjected to first-line DST, from which 423 (35.8%) were classed as MDR-TB isolates. Despite the availability of FQ and SLID DST data for 48 and 9 MDR-TB isolates from Brazil and Guinea-Bissau, respectively, XDR-TB isolates were only detected in the Portuguese dataset (n = 40) comprising 40.8% of the MDR-TB isolates tested for susceptibility to FQ and SLID in this country. This rate agrees with previous data on this setting and is the cause of major concern regarding ongoing extension of drug resistance to third-line drugs (Perdigao et al., 2013; Perdigao et al., 2016). In the Brazil dataset, no XDR-TB isolate was detected which, is not unexpected given the low number of isolates with data for second-line drug susceptibility testing (n = 48) and is in line with the low rate of XDR-TB estimated by the WHO for Brazil (5.9%) (World Health Organization, 2016).

**Table 1:** Sample characteristics: datasets included in the present study by country of origin and stratification of the number of isolates by isolation year, susceptibility testing and drug resistance.

Country of origin	Isolation year																	No. of isolates with DST (first/s line)	No. of drug resistant isolates (%)						
	1995	1996	1997	1998	2003	2005	2006	2007	2008	2009	2010	2011	2012	2013	2014	2015	2016		INH	RIF	STR	EMB	PZA	MDR	XDR
Angola (n = 89)	0	0	0	0	0	0	0	0	0	0	0	0	0	0	89	0	0	89/0	17 (19.1)	6 (6.7)	13 (14.6)	5 (5.6)	5 (5.6)	5 (5.6)	–
Brazil (n = 964)	0	0	0	0	0	0	50	65	37	38	71	123	202	161	149	46	22	713/48	386 (54.1)	275 (38.6)	67 (15.6)	29 (6.7)	4 (4.4)	269 (37.7)	0 (0)
Guinea-Bissau (n = 13)	0	0	0	0	0	0	0	0	0	0	0	0	13	0	0	0	0	13/9	9 (69.2)	9 (69.2)	6 (46.2)	7 (53.8)	7 (53.8)	9 (69.2)	0 (0)
Mozambique (n = 14)	0	0	0	0	0	0	0	0	0	0	0	0	0	14	0	0	0	0/0	–	–	–	–	–	–	–
Portugal (n = 367)	1	1	5	2	2	3	3	36	91	114	42	35	10	4	7	10	1	367/98	174 (47.4)	67 (15.6)	150 (40.9)	97 (26.4)	112 (31.2)	140 (38.1)	40 (40.8)
Total (n = 1447)	1	1	5	2	2	3	53	101	128	152	113	158	225	179	245	56	23	1182/155	586 (49.6)	434 (36.7)	236 (26.3)	138 (15.4)	128 (23.2)	423 (35.8)	40 (25.8)

The convenience-nature of the sampling constitutes a limitation to this study, but this is not an untypical design in a pathogen genetic setting, where clinically and epidemiological relevant isolates are characterized. Isolate inclusion based on availability of genotypic data is a common criterion due to their association with drug resistance. The prevalence of drug resistant strains does not necessarily reflect their local prevalence, except in the Angolan dataset, which was systematically collected over a defined period to assess drug resistance prevalence in a specific study population (Perdigao et al., 2017). Nonetheless, the datasets herein combined comprise the basis for the first attempt to investigate the global molecular epidemiology within the CPLP, enabling the investigation of possible links, and, provide a considerable progress to an expanding body of knowledge concerning *M. tuberculosis* genetic diversity in its member-states. It is, however, necessary to bear in mind potential bias in low sampled settings such as Mozambique and Guinea-Bissau and, furthermore, we stress the importance of future prospective studies that can play a key role in obtaining increasingly robust estimates on drug resistance in countries that do not carry out DST on a routine basis and, evaluate the impact of control programme interventions. Such studies should ideally encompass a uniform DST methodology, both country-wide and CPLP-wide, based on rapid nonradiometric liquid culture (Bemer et al., 2002). Currently, lack of routine culture-based DST poses an enormous limitation to this study and such future studies. Country policy on culture-based DST is not uniform across the CPLP where: Portugal performs it routinely for all culture-positive cases; CPLP African countries due to the high number of cases and lack of adequate laboratory support do not perform it routinely; and, in Brazil, country policy mandates routine culture-based DST to be carried out mainly for HIV co-infection cases, relapse, retreatment or treatment default cases. Herein, a subset of the samples obtained from Brazil were subjected to DST using REMA which should also be considered when analysing the data, albeit the good correlation between both methods on the evaluation of susceptibility to first-line drugs (Coban et al., 2014; Palomino et al., 2002).

### 3.2 Assessing *M. tuberculosis* population structure diversification through spoligotyping

To uncover the *M. tuberculosis* population structure in a country-wise manner, the combined dataset included spoligotyping data for 1239 clinical isolates, which corresponded to: 89 (7.2%) isolates from Angola; 760 (61.3%) from Brazil; 13 (1.0%) from Guinea-Bissau; 14 (1.1%) from Mozambique; and, 363 (29.3%) from Portugal. A total of 248 spoligotyping profiles were obtained, which corresponded to 164 SITs and 62 profiles classified as Orphan (Supplementary Tables S1 and S2). Additionally, 22 new SITs were identified upon data submission to SITVITEXTEND (SIT4139–4160). Overall, the most prevalent SITs found were SITs 20/LAM1 (n = 157; 12.7%), 42/LAM9 (n = 94/7.6%) and 53/T1 (n = 92; 7.4%) while the most prevalent clades encountered were the T1 (n = 211; 17.0%), LAM1 (n = 205; 16.5%), LAM9 (n = 141; 11.4%), H1 (n = 90; 7.3%), H3 (n = 83; 6.7%) and LAM4 (n = 67; 5.4%).

Comparing the distribution of the spoligotyping profiles by country, only the Portuguese dataset included isolates from all major *M. tuberculosis* lineages (Table 2). Remarkably, no Beijing strain was encountered on the Brazilian dataset despite its the large sample size. This is most likely correlated with the low prevalence of Beijing strains in this country as only 10 (0.3%) out of 2992 strains deposited in SITVITWEB belonged to the Beijing clade (Demay et al., 2012). The prevalence in Rio Grande do Sul state, from where the Brazilian dataset originates, is most likely even lower given the fact no Beijing isolate was identified in this study (Table 2). Previously reported data in the literature support this low prevalence of Beijing strains among the *M. tuberculosis* populational structure in Brazil (<1%) and, similarly, across South American countries such as Argentina, Paraguay and Chile (Balcells et al., 2015; Demay et al., 2012; Gomes et al., 2012). An exception is Peru that has reported a prevalence of Beijing strains ranging between 5.5 and 9.15% (7.4% on SITVITWEB) (Caceres et al., 2014; Demay et al., 2012; Sheen et al., 2013). In contrast, in Guinea-Bissau, despite the reduced sample size, 8 (61.5%) out of 13 isolates belonged to Beijing clade. Although this fact is not surprising since Beijing isolates were previously detected in this country, the number of isolates retrieved from such a low-sized sample suggests that Beijing isolates may have been introduced more recently and appear to be emerging in this setting, as previously suggested (Rabna et al., 2015). Such emergence of Beijing strains beyond what can be considered its normal phylogeographical niche may be associated and being driven by (multi)drug resistance and/or HIV co-infection, a phenomenon previously reported in other settings (Lasunskaia et al., 2010; Liu et al., 2018; Viegas et al., 2013).

**Table 2:** – Isolates characterized by spoligotyping, number of obtained SITs and number of isolates classified in the main spoligotyping lineages per dataset.

Country	No. of isolates	No. of SITs	No. of orphan profiles	Spoligotyping main lineages - no. of isolates (%)							
				Beijing	CAS	EAI	H	LAM	S	T	X
Angola	89	25	8	0 (0)	0 (0)	0 (0)	1 (1.1)	55 (61.8)	0 (0)	31 (34.8)	0 (0)
Brazil	760	90	81	0 (0)	0 (0)	1 (0.1)	152 (20)	326 (42.9)	8 (1.1)	185 (24.3)	13 (1.7)
Guinea-Bissau	13	5	0	8 (61.5)	0 (0)	0 (0)	0 (0)	2 (15.4)	0 (0)	0 (0)	0 (0)
Mozambique	14	6	4	0 (0)	0 (0)	2 (14.3)	3 (21.4)	2 (14.3)	2 (14.3)	2 (14.3)	0 (0)
Portugal	363	77	13	26 (7.2)	4 (1.1)	4 (1.1)	18 (5)	226 (62.3)	3 (0.8)	42 (11.6)	17 (4.7)

**Fig. 1:**
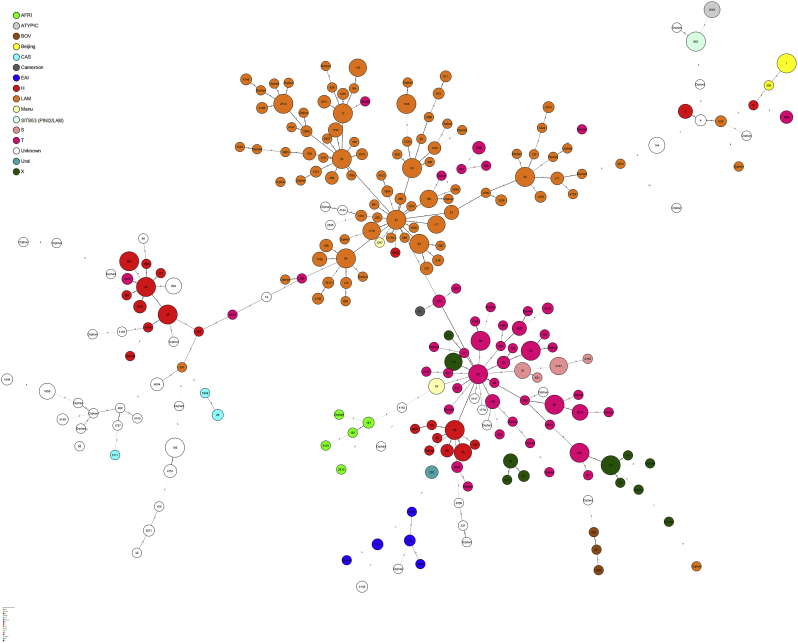
Minimum Spanning Tree (MST) based on all available spoligotyping patterns in this study (n = 1239 isolates). Node coloring is shown in function of lineages and thickness and/or shape of branches (continuous, dashed, dotted, black or gray) varied in function of spacer changes between patterns. The number of changes is indicated on branches.

A common denominator appears to be LAM and the “ill-defined” T clade, found to be present across all datasets with diverging prevalence, highlighting specific populational structures across all settings (Table 2). Analysis of a minimum spanning tree based on spoligotypes (Fig. 1) reveals that LAM lineage is particularly diverse in this study. Interestingly, while the prevalence of LAM strains in the Angolan and Portuguese datasets was similar, there was a difference in the distribution of the most prevalent SITs (>5%) among LAM clades in the Portuguese and Angolan datasets (Table 3). Angola showed a prevalence of SIT42/LAM9 strains that was twice the one in the Portuguese dataset, while the latter had a higher prevalence of SIT20/LAM1 strains. Also, SIT1106/LAM4 strains were only detected in Portugal, stressing the endemicity of this clade and its most likely origin in this country (Supplementary Fig. S1). Although previously reported in Brazil, the number of SIT1106/LAM4 isolates herein included from Portugal exceeds by 1.7-fold the number of these strains present in the SITVIT database (Table 3). Moreover, SIT1106/LAM4 have been associated with M/XDR-TB isolates of the Q1 cluster in Portugal and its global importance has recently been highlighted due to the potential to extend its drug resistance profile towards third-line drugs (Perdigao et al., 2016).

**Table 3:** Comparative prevalence of the main SITs found, herein defined as SITs with a prevalence equal or >5.0% in at least one dataset, associated clade and distribution per dataset.

SIT/Clade	Spoligotyping profile	No. of isolates (%)					Distribution in countries with ≥ 2% of a given SIT^a^
		Angola	Brazil	Guinea-Bissau	Mozambique	Portugal	
1/Beijing		0 (0)	0 (0)	8 (61.5)	0 (0)	25 (6.9)	CN = 19.12, US = 18.89, JP = 10.86, ZA = 7.81, RU = 6.6, VN = 3.67, IN = 3.56, PE = 2.96, MY = 2.79
20/LAM1		17 (19.1)	43 (5.7)	0 (0)	0 (0)	97 (26.7)	BR = 17.62, US = 17.08, HT = 7.3, NA = 6.66, PT = 5.26, FR = 5.26, VE = 4.51, ZA = 2.9, ES = 2.69, AR = 2.36, PE = 2.26, CO = 2.15, BE = 2.04
34/S		0 (0)	0 (0)	0 (0)	2 (14.3)	3 (0.8)	ZA = 17.11, US = 14.89, IT = 11.78, CA = 8.22, BR = 8.00, FR = 4.00, BG = 3.44, HT = 2.78, BE = 2.33
42/LAM9		15 (16.9)	46 (6.1)	2 (15.4)	0 (0)	31 (8.5)	BR = 14.45, US = 10.6, CO = 7.64, MA = 6.3, IT = 5.85, FR = 4.54, AR = 3.53, PE = 3.31, ES = 2.99, VE = 2.96, ZA = 2.83, HT = 2.16, RU = 2.06
45/H1		0 (0)	57 (7.5)	0 (0)	0 (0)	1 (0.3)	AT = 16.51, MQ = 11.01, US = 9.17, CO = 8.26, IT = 8.26, BR = 8.26, GP = 7.34, DE = 3.67, NL = 2.75, LC = 2.75, SE = 2.75
50/H3		0 (0)	56 (7.4)	0 (0)	0 (0)	3 (0.8)	US = 14.2, PE = 12.6, BR = 7.85, FR = 5.56, AT = 4.92, ES = 4.39, IT = 4.39, ZA = 3.27, AR = 3.08, CM = 3.01, CZ = 2.96, HT = 2.77, TR = 2.58, SE = 2.29
53/T1		12 (13.5)	59 (7.8)	0 (0)	2 (14.3)	19 (5.2)	US = 11.96, FR = 7.14, BR = 5.87, IT = 4.82, TR = 4.51, ZA = 4.39, PE = 4.29, AT = 3.09, CN = 2.82, FI = 2.65, MX = 2.58, ET = 2.35, SA = 2.26, AR = 2.19, ES = 2.09
65/T1		0 (0)	60 (7.9)	0 (0)	0 (0)	0 (0)	BR = 39.29, HT = 21.43, US = 9.29, FR = 6.43, ES = 3.57, AT = 2.86, CM = 2.14
129/EAI6-BGD		0 (0)	0 (0)	0 (0)	1 (7.1)	1 (0.3)	MZ = 26.15, BR = 26.15, GF = 12.31, US = 7.69, MW = 6.15, ZA = 4.62, TN = 3.08
144/Unknown		5 (5.6)	0 (0)	0 (0)	0 (0)	0 (0)	GM = 29.51, BE = 26.23, FR = 13.11, BG = 9.84, US = 6.56, IT = 3.28
181/AFRI_1		0 (0)	0 (0)	1 (7.7)	0 (0)	1 (0.3)	GW = 34.03, GM = 34.03, US = 7.29, FR = 5.56, IT = 4.51, NL = 3.82, GB = 2.78, SN = 2.43
187/AFRI_1		0 (0)	0 (0)	1 (7.7)	0 (0)	0 (0)	GW = 47.47, GM = 18.18, FR = 11.11, US = 11.11, TN = 5.05, BE = 2.02
244/T1		6 (6.7)	2 (0.3)	1 (7.7)	0 (0)	6 (1.7)	BR = 23.4, ZA = 13.48, PT = 11.35, BD = 9.93, FR = 9.22, ZM = 5.67, GW = 4.96, TZ = 4.26, US = 3.55, MZ = 2.13
806/EAI1-SOM		0 (0)	0 (0)	0 (0)	1 (7.1)	1 (0.3)	ZA = 44.44, MZ = 31.48, US = 14.81, ZW = 3.70, NO = 3.70
811/LAM11-ZWE		0 (0)	0 (0)	0 (0)	1 (7.1)	1 (0.3)	ZA = 40.35, MZ = 26.32, ZW = 14.04, US = 8.77, ZM = 3.51, BE = 3.51, TZ = 3.51
1106/LAM4		0 (0)	0 (0)	0 (0)	0 (0)	38 (10.5)	PT = 40.91, BR = 31.82, ES = 18.18, IT = 4.55, PE = 4.55
2375/H1		0 (0)	0 (0)	0 (0)	3 (21.4)	0 (0)	ZA = 50.00, PE = 21.43, BE = 14.29, MZ = 7.14, DE = 7.14
4140/Unknown		0 (0)	0 (0)	0 (0)	2 (14.3)	0 (0)	New SIT

a Countrywide distribution is only shown for SITs with ≥2% of a given SIT as compared to their total number in the SITVIT database; the 2 letter country codes are according to http://en.wikipedia.org/wiki/ISO_3166-1_alpha-2:; AD Andorra; AE United Arab Emirates; AF Afghanistan; AG Antigua and Barbuda; AI Anguilla; AL Albania; AM Armenia; AO Angola; AQ Antarctica; AR Argentina; AS American Samoa; AT Austria; AU Australia; AW Aruba; AX Åland Islands; AZ Azerbaijan; BA Bosnia and Herzegovina; BB Barbados; BD Bangladesh; BE Belgium; BF Burkina Faso; BG Bulgaria; BH Bahrain; BI Burundi; BJ Benin; BL Saint Barthelemy; BM Bermuda; BN Brunei; BO Bolivia; BQ Bonaire; Sint Eustatius and Saba; BR Brazil; BS Bahamas; BT Bhutan; BV Bouvet Island; BW Botswana; BY Belarus; BZ Belize; CA Canada; CC Cocos (Keeling) Islands; CD Congo, the Democratic Republic of the; CF Central African Republic; CG Congo; CH Switzerland; CI Ivory Coast; CK Cook Islands; CL Chile; CM Cameroon; CN China; CO Colombia; CR Costa Rica; CU Cuba; CV Cabo Verde; CW Curaçao; CX Christmas Island; CY Cyprus; CZ Czech Republic; DE Germany; DJ Djibouti; DK Denmark; DM Dominica; DO Dominican Republic; DZ Algeria; EC Ecuador; EE Estonia; EG Egypt; EH Western Sahara; ER Eritrea; ES Spain; ET Ethiopia; FI Finland; FJ Fiji; FK Falkland Islands (Malvinas); FM Micronesia, Federated States of; FO Faroe Islands; FR France; GA Gabon; GB United Kingdom of Great Britain and Northern Ireland; GD Grenada; GE Georgia; GF French Guiana; GG Guernsey; GH Ghana; GI Gibraltar; GL Greenland; GM Gambia; GN Guinea; GP Guadeloupe; GQ Equatorial Guinea; GR Greece; GS South Georgia and the South Sandwich Islands; GT Guatemala; GU Guam; GW Guinea-Bissau; GY Guyana; HK Hong Kong; HM Heard Island and McDonald Islands; HN Honduras; HR Croatia; HT Haiti; HU Hungary; ID Indonesia; IE Ireland; IL Israel; IM Isle of Man; IN India; IO British Indian Ocean Territory; IQ Iraq; IR Iran; IS Iceland; IT Italy; JE Jersey; JM Jamaica; JO Jordan; JP Japan; KE Kenya; KG Kyrgyzstan; KH Cambodia; KI Kiribati; KM Comoros; KN Saint Kitts and Nevis; KP Korea, Democratic People's Republic of (North Korea); KR Korea, Republic of (South Korea); KW Kuwait; KY Cayman Islands; KZ Kazakhstan; LA Laos; LB Lebanon; LC Saint Lucia; LI Liechtenstein; LK Sri Lanka; LR Liberia; LS Lesotho; LT Lithuania; LU Luxembourg; LV Latvia; LY Libya; MA Morocco; MC Monaco; MD Moldova, Republic of; ME Montenegro; MF Saint Martin (French part); MG Madagascar; MH Marshall Islands; MK Macedonia; ML Mali; MM Myanmar; MN Mongolia; MO Macao; MP Northern Mariana Islands; MQ Martinique; MR Mauritania; MS Montserrat; MT Malta; MU Mauritius; MV Maldives;  MW Malawi; MX Mexico; MY Malaysia; MZ Mozambique; NA Namibia; NC New Caledonia; NE Niger; NF Norfolk Island; NG Nigeria; NI Nicaragua; NL Netherlands; NO Norway; NP Nepal; NR Nauru; NU Niue; NZ New Zealand; OM Oman; PA Panama; PE Peru; PF French Polynesia; PG Papua New Guinea; PH Philippines; PK Pakistan; PL Poland; PM Saint Pierre and Miquelon; PN Pitcairn; PR Puerto Rico; PS Palestine (Palestinian Territory, Occupied - Consists of the West Bank and the Gaza Strip); PT Portugal; PW Palau; PY Paraguay; QA Qatar; RE Réunion; RO Romania; RS Serbia; RU Russian Federation (Russia); RW Rwanda; SA Saudi Arabia; SB Solomon Islands; SC Seychelles; SD Sudan; SE Sweden; SG Singapore; SH Saint Helena, Ascension and Tristan da Cunha (Saint Helena); SI Slovenia; SJ Svalbard and Jan Mayen; SK Slovakia; SL Sierra Leone; SM San Marino; SN Senegal; SO Somalia; SR Suriname; SS South Sudan; ST Sao Tome and Principe; SV El Salvador; SX Sint Maarten (Dutch part); SY Syrian Arab Republic; SZ Swaziland; TC Turks and Caicos Islands; TD Chad; TF French Southern Territories (Terres australes françaises); TG Togo; TH Thailand; TJ Tajikistan; TK Tokelau; TL Timor-Leste (East Timor); TM Turkmenistan; TN Tunisia; TO Tonga; TR Turkey; TT Trinidad and Tobago; TV Tuvalu; TW Taiwan; TZ Tanzania; UA Ukraine; UG Uganda; UM United States Minor Outlying Islands; US United States of America; UY Uruguay; UZ Uzbekistan; VA Holy See; VC Saint Vincent and the Grenadines; VE Venezuela; VG Virgin Islands (British); VI Virgin Islands (US); VN Vietnam; VU Vanuatu; WF Wallis and Futuna; WS Samoa; YE Yemen; YT Mayotte; ZA South Africa; ZM Zambia; ZW Zimbabwe.

Despite detected in Portugal and Brazil, EAI strains appear to be of particular epidemiological significance in Mozambique where, despite a low sample size, 4 out of 14 (28.6%) strains belonged to this lineage (Table 2). This high prevalence is likely related with the proximity to the Indian subcontinent (Gagneux et al., 2006). In fact, the newly described SIT4140 could be specific to Mozambique and although labelled as an Unknown clade due to the absence of specific spacers it could potentially belong to the EAI lineage. In Brazil, Haarlem strains (SIT45/H1 and SIT50/H3, mostly) are also noteworthy, possibly representing more recently introduced European strains brought by European settlers of diverse origins (e.g., Italy, Poland, Germany and Russia) over two centuries ago (Garzelli et al., 2010; Jagielski et al., 2010; Lari et al., 2007). Still in the Brazilian dataset, the prevalence of T strains appears to be mostly underpinned by two different clades: SIT53/T1 and SIT65/T1. While SIT53/T1 strains are well dispersed through CPLP countries and was detected in all datasets except Guinea-Bissau, SIT65/T1 shows a much more restricted distribution and is in fact more prevalent in Brazil, with our data supporting a much higher prevalence in Rio Grande do Sul state where we obtained a prevalence of 7.9% (Table 3). Finally, 14 out of the 17 main SITs (prevalence ≥5%) detected in each country correlate well with the countries associated with at least 2% of the SIT's global distribution, thus strengthening the current understanding of *M. tuberculosis* phylogeography (Table 3).

### 3.3 A MIRU-VNTR framework for the CPLP

Of the 999 (69.0%) isolates genotyped by MIRU-VNTR, 181, 420 and 398 isolates were genotyped by 12, 15 and 24-loci MIRU-VNTR, respectively (Table 4). The MIRU-VNTR loci set used varied according to practice of the laboratories participating in this study. It was not possible to obtain data on MIRU-VNTR typing from the Mozambique dataset.

**Table 4:** Number of isolates genotyped by 12, 15 and 24-loci MIRU-VNTR.

MIRU-VNTR set	No. of isolates (%)					Total
	Angola	Brazil	Guinea-Bissau	Mozambique	Portugal	
12-loci	0 (0)	181 (100)	0 (0)	0 (0)	0 (0)	181
15-loci	0 (0)	420 (100)	0 (0)	0 (0)	0 (0)	420
24-loci	89 (22.4)	83 (20.9)	13 (3.3)	0 (0)	213 (53.5)	398

To understand the local transmission patterns and assess strain identity using a method with a higher discriminative power, we started by considering all clinical isolates genotyped by 15-loci MIRU-VNTR. The latter, can also be obtained from strains genotyped using the 24-loci set and has shown a comparative discriminatory power when compared with the 15-loci set. From the possible 818 MIRU-VNTR profiles available, 15 were excluded due to incomplete profiles, rendering a total of 802 15-loci MIRU-VNTR profiles suitable for analysis. Upon construction of a MIRU-VNTR dendrogram, we have identified 522 unique profiles and 75 genetic clusters encompassing a total of 356 (44.3%) clinical isolates (Supplementary Fig. S2). The remaining 447 (55.7%) isolates were classified as non-clustered (NC) isolates. Combining both country of origin and the genotypic profile generated by 15-loci MIRU-VNTR, 65 clusters were country-specific while the remaining 10 clusters were defined as cross-border clusters (CPLP clusters), containing between 2 and 17 isolates each from distinct countries. The largest CPLP cluster (CPLP-01) included 17 *M. tuberculosis* isolates from three distinct countries: Angola (n = 9), Brazil (n = 6) and Portugal (n = 2) (Supplementary Table S1).

However, despite the high discriminatory power (HGI = 0.993), MIRU-VNTR was not fully congruent with cluster associated SITs as 21 of 51 clusters that had two or more isolates showed mixed spoligotyping profiles (Supplementary Table S3). In nine of these, clustering incongruences cannot be simply explained by subsequent diversification at the DR locus level, suggesting convergence of MIRU-VNTR profiles.

**Fig. 2:**
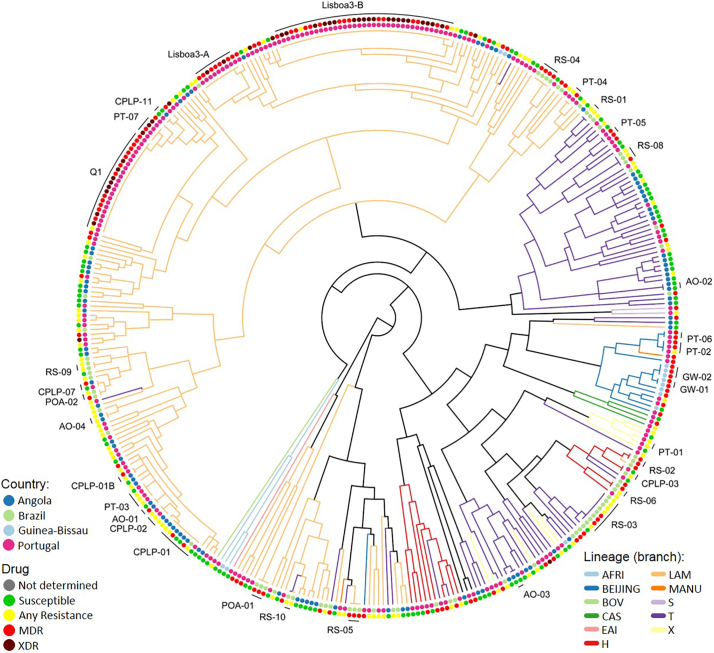
Twenty-four loci MIRU-VNTR tree obtained for 381 isolates annotated with color-coded tip points that represent the country of origin (inner rim) and drug resistance (outer rim). Six cross-border clusters can be observed in the present tree as well as the association of XDR-TB isolates in Portugal with two main MDR-TB genetic clusters (Lisboa3-B and Q1).

Next, to compare with 24-loci MIRU-VNTR clustering, from the 398 isolates genotyped by this method, 17 failed to amplify at least one locus and a dendrogram was constructed for the remaining 381 isolates: 89 from Angola, 71 from Brazil, 13 from Guinea-Bissau and 208 from Portugal (Fig. 2, Supplementary Fig. S3). Using this method, we have identified 33 clusters encompassing a total of 152 (40.2%) clinical isolates (HGI = 0.986). The lower discriminatory power obtained was mostly due to the high prevalence of MDR- and XDR-TB clones in the Portuguese dataset and because, most of the susceptible isolates from Brazil were genotyped by 15-loci MIRU-VNTR whereas drug resistant isolates were more likely to have been genotyped by 24-loci MIRU-VNTR. Nevertheless, some clusters were further resolved leading to the definition of two additional clusters but supporting at least partial clustering of 32 of the original 75 genetic clusters defined using the 15-loci set (Supplementary Table S4). A subset of 6 clusters defined using the 24-loci set yields further support to clonal expansion at a transnational level. A MIRU-VNTR dendrogram including all isolates genotyped by 15 or 24-loci MIRU-VNTR is also included as Supplemental material (Supplementary Fig. S4).

Beyond the Lusophone space, and concerning the Beijing strains isolated from Guinea-Bissau, analysis by 24-loci allows the assignment of three isolates to type #94-32 (MLVA MtbC15-9 scheme), also termed as Russian/Asian clone CC1 (IS6110-RFLP-defined A0-cluster) (Merker et al., 2015; Mokrousov et al., 2008). These findings place the most likely origin of these strains in the former Soviet Union as type #94–32 is one of the two main Beijing types in Russia and highly prevalent in the former soviet Central Asia (Merker et al., 2015; Mokrousov et al., 2018; Mokrousov et al., 2008; Skiba et al., 2015).

**Fig. 3:**
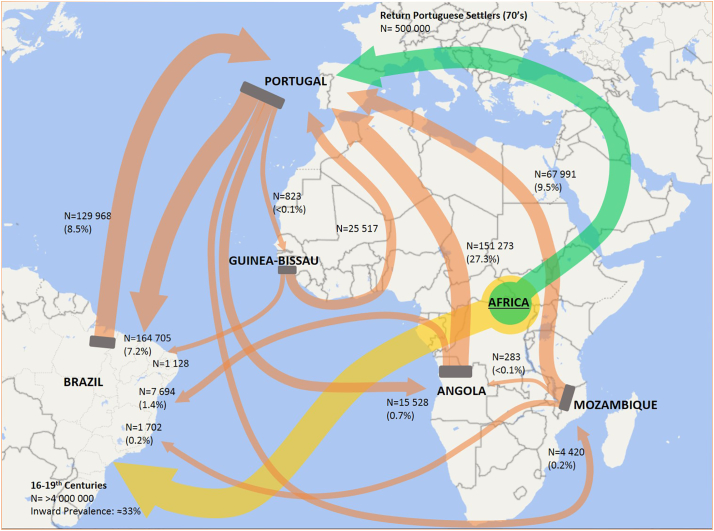
Lusophone migratory system depicting global migratory flows between the CPLP countries herein studied (orange arrows). Total migratory flow is indicated and proportionally represented by each arrow connecting these countries/regions. The percentage values indicated within parentheses represent the fraction of the total outward migratory flow from the country of origin. It is noteworthy the role played by Portugal as a central hub in this migratory system and the intense migratory events occurring between Portugal, Angola and Brazil. Part of the African diaspora is also represented in the present map as a major mass migratory event, taking place over four centuries, connecting the African continent with Brazil and leading to the introduction of at least 4,000,000 African slaves (yellow arrow). Another more recent and historical mass migratory event, with potential impact on the Portuguese *M. tuberculosis* populational structure in Portugal, occurred during the 70's as the result of the return of Portuguese settlers following the end of the Portuguese Colonial War (green arrow). Source: Trends in International Migrant Stock: Migrants by Destination and Origin. United Nations, Department of Economic and Social Affairs, 2015; figure generated using Microsoft® PowerPoint® 2016 [Version 1707] and Microsoft® Excel® 2016 (Version 1707), incl. Microsoft® Power Map 3D Data Visualization Tool (https://products.office.com/en-us/business/office). (For interpretation of the references to color in this figure legend, the reader is referred to the web version of this article.)

The potential epidemiological links and similar population structures between present Lusophone countries may have its roots back to the 16th century with the historical overseas exploration period that brought together diverse cultures across distinct continents. More specifically, the similar population structure may have its common ground in the transatlantic trade routes and the African diaspora linking Portugal, Brazil and probably having Central Africa, a region that presently includes Angola, as its hub (Patarra and Fernandes, 2011). It is estimated that until 1850, slave trade introduced about 4 million African people in Brazil, most likely shaping the present *M. tuberculosis* population structure of this country since this population may have reached up to one-third of the total population (Graham, 2002; Prado Jr., 2012). Furthermore, the importance of this population as an incoming TB reservoir is well documented from archaeological specimens (Jaeger et al., 2013; Jaeger et al., 2012). However, indistinguishable genetic clusters probably have its origins in more recent migratory events, difficult to ascertain due to the complexity of the Lusophone migratory system (Fig. 3), which some consider to be structured around three main hubs: Angola, Brazil and Portugal (Baganha, 2009; Marques and Góis, 2011). More recent periods of migration during the third, and early fourth, quarter of the 20th century, after the independence of Angola led to the return of approximately 500,000 settlers from former Portuguese colonies to Portugal, mostly coming from Angola, and a small minority to Brazil (Baganha, 2009). This mass migration might have, as well, played a key role in shaping *M. tuberculosis* population structure in recent times and underpin some of the cross-border clusters here observed. Nevertheless, these considerations do not exclude the ongoing migratory flows that take place up to this day and that might also explain smaller or still undetected clusters. Also, the detection of other unusual underrepresented clades are likely associated with more recent events leading to the introduction and emergence of these strains or, with additional migratory systems to which each country is prone to be inserted in given the local geographical context (Goldblatt et al., 2014; Mokrousov, 2016; Tolentino et al., 2011).

### 3.4 MIRU-VNTR loci and allelic diversities

**Fig. 4:**
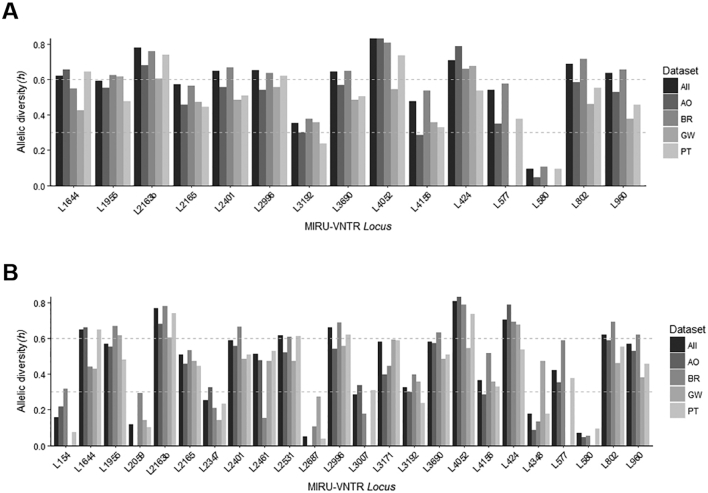
Allelic diversities obtained for the sample subsets genotyped with 15 (A) and 24 (B) loci MIRU-VNTR. Allelic diversities for the combined datasets or for each individual dataset are shown. Horizontal dashed lines are used to highlight the breakpoints between low-to-intermediate and intermediate-to-high allelic diversities.

Concerning the discriminatory capability of the different MIRU-VNTR loci used, we calculated the allelic diversity (*h*) for each locus, overall and across each country set, for the two sets of strains assessed by 15 and 24-loci MIRU-VNTR (Fig. 4). MIRU-VNTR loci were classified as poorly (*h* < 0.3), moderately (*h* = 0.3–0.6), and highly (*h* > 0.6) discriminatory. Globally, *loci* 2163b and 4052 showed the highest discriminatory power (h = 0.77 and 0.81, respectively) while loci 580 and 2687 present the lowest allelic diversity (h = 0.07 and 0.05, respectively). The allelic diversity scores found for this sample of isolates were comparable to other published datasets comprised by clinical isolates of diverse geographical origin, except for loci 580 and 2687 (Mokrousov et al., 2004; Sola et al., 2003; Supply et al., 2006). These loci exhibited very low allelic diversities across all datasets that strikingly contrast with the ones obtained in the aforementioned studies. However, when comparing with studies of a more geographically localized scope, the allelic diversities obtained herein tend to be higher (Chen et al., 2012; Cowan et al., 2002; Mazars et al., 2001; Mokrousov et al., 2004; Shamputa et al., 2010; Supply et al., 2003; Wada et al., 2009).

### 3.5 Assigning phylogenetic defined sub-lineages to LAM strains

Given the epidemiological importance of the LAM lineage in the CPLP, we attempted to assign Spoligotyping and Region of Difference(RD)-based LAM-sublineages to the isolates genotyped by 24-loci MIRU-VNTR as the tree obtained for these isolates is more robust from a phylogenetic standpoint (Supplementary Fig. 2). The resulting tree was examined for the distribution of specific MIRU-VNTR alleles previously proposed by Mokrousov et al. (2016) as a proxy for some LAM-sublineages (Mokrousov et al., 2016).

Within the LAM lineage, isolates bearing the 802_1 allele (locus 802, number of repeats: 1) which was proposed to correlate with RD174 sublineage were found to comprise a monophyletic branch containing 184 isolates in which all, but 1 isolate, exhibit this specific allele. This sub-lineage harbours the most important strains from an epidemiological standpoint, specifically: the Lisboa3-A/B (SIT20/LAM1) or Q1 (SIT1106/LAM4) clusters in Portugal; RS-04 (SIT106/LAM7) cluster in Brazil; or, the CPLP-1 cluster that is shared at a transnational level. Supporting this, data on the genome-wide analysis of Lisboa3 and Q1 strains have already shown the association between these clusters and RD174/RD^RIO^ (Perdigao et al., 2014).

Thirty-one isolates were also found to form a monophyletic branch containing the 154_1 allele as a marker for the RD115 sublineage. This branch included, among others, the “*pinnipedii*-like” strains SIT863/PINI2 which has been shown by some of us to in fact belong to the LAM lineage (Dalla Costa et al., 2015). Interestingly, the SIT863/PINI2 strains underwent diversification at locus 154 (154_2). The phylogenetic positioning of SIT863/PINI2 in the RD115-sublineage is congruent with previous data on its SNP barcode lineage (4.3.3) as a RD115 sub-lineage branch (Coll et al., 2014; Dalla Costa et al., 2015). Nevertheless, some phylogenetic incongruences were noted as other unrelated strains, e.g., Beijing or T1, were included in this branch, thus exemplifying the problem of MIRU-VNTR convergence.

The “SIT33-sublineage” was also identifiable among LAM strains: characterized by MIRU-VNTR alleles 2401_2 and 3171_1, a branch was identified as associated with these alleles. This specific branch was composed by 18 isolates of which 4 belonged to SIT33/LAM3 while four exhibited putative SIT33-derived spoligotyping profiles. Three isolates were, nevertheless, characterized by spoligotyping profiles apparently unrelated to SIT33. Furthermore, MDR-TB RS-04 cluster (SIT106/LAM7) was found to belong to this sub-lineage highlighting its importance in Brazil.

Additionally, we also examined the tree for allele 2059_1 which is proposed to be associated with the SIT388-sublineage, which was not found for any LAM isolate except for a branch within the RD115 sublineage. This marker has been reported as the least robust concerning its Positive Predictive Value but interestingly, this putative sub-branch comprised five isolates, aside an isolate with the 2059_2 allele, that included the SIT863/PINI2 isolates as the only strains bearing a profile compatible with descent from SIT388. However, SIT388-sublineage is proposed by Mokrousov et al. (2016) to be derived directly from SIT42/LAM9/RD115 strains and therefore constitute a Japan-endemic parallel branch to the RD115 sublineage consistent with its association with SNP barcode 4.3.1 (Coll et al., 2014; Mokrousov et al., 2016).

### 3.6 Drug resistance association with MIRU-VNTR clusters and spoligotyping clades

We have further analysed the distribution of drug resistant isolates across the MIRU-VNTR dendrograms and SITs/clades. Intersecting phenotypic drug resistance with the previously defined 24-loci MIRU-VNTR generated clusters, 30 where associated with drug resistant strains. From these, 22 and 4 comprised MDR-TB and XDR-TB strains, respectively. Looking further into the association of these clusters with drug resistance, it is noteworthy the statistically significant higher proportion of XDR-TB within Lisboa3, Q1 and Q1-derived PT-07 (SIT1106/LAM4) clusters in comparison to MDR-TB and/or strains exhibiting any type of drug resistance other than MDR/XDR in the same clusters (z-test, p < 0.05). In Lisboa3 cluster this association was also significant when comparing the higher proportion of MDR-TB strains with the proportion of strains exhibiting any type of drug resistance (z-test, p = 0.02).

Finally, the proportion of non-clustered pan-susceptible isolates was significantly higher than non-clustered isolates exhibiting MDR, XDR or any type of drug resistance (z-test, p < 0.01).

When analysing the dendrogram generated using 15-loci set, which enables the use of a larger subset of the isolates herein analysed, the statistical association is further extended to the clusters PT-03, showing a higher proportion of isolates with any type of drug resistance other than MDR/XDR relative to susceptible isolates (z-test, p = 0.019); and, cluster RS-05, showing a higher proportion of MDR-TB than susceptible isolates (z-test, p = 0.021).

This data provides further support to the epidemiological importance of Lisboa3 and Q1 phylogenetic clades that, despite the inclusion of other datasets from diverse countries, maintain a high degree of endemicity in Portugal and, calls for the adoption of specific control measures (e.g., active case finding) focused on the prevention of transmission of these highly resistant strains. Moreover, these two clades (MtbC15-9 types: 5298-54 [Lisboa3-B] and 10,571-67 [Q1], respectively) have an increasing importance in the European context as these have been recently identified and shortlisted as MDR-TB associated European cross-border clusters, with likely origin in Portugal, through the detection in the United Kingdom (Q1, n = 1) and France (Lisboa3-B, n = 1).(European Centre for Disease Prevention and Control/WHO Regional Office for Europe, 2017).

These cluster level associations were also reflected by specific associations found between the spoligotyping clade and resistance type: a higher proportion of susceptible strains, in comparison to isolates displaying MDR, were found among the H1 (z-test, p < 0.001), LAM3 (z-test, p = 0.002) and LAM9 (z-test, p = 0.024) clades; MDR isolates were found in a higher proportion, in comparison to susceptible isolates, in H3 (z-test, p < 0.001), LAM5 (z-test, p < 0.001), LAM4 (z-test, p < 0.001) and PINI2 (z-test, p = 0.003) clades; and, XDR-TB isolates were found in a higher proportion among LAM1 (z-test, p < 0.001) and LAM4 (z-test, p < 0.001) isolates when compared to MDR or susceptible isolates.

### 3.7 CPLP-TB: a new web-based tool for strain tracking across the CPLP

**Fig. 5:**
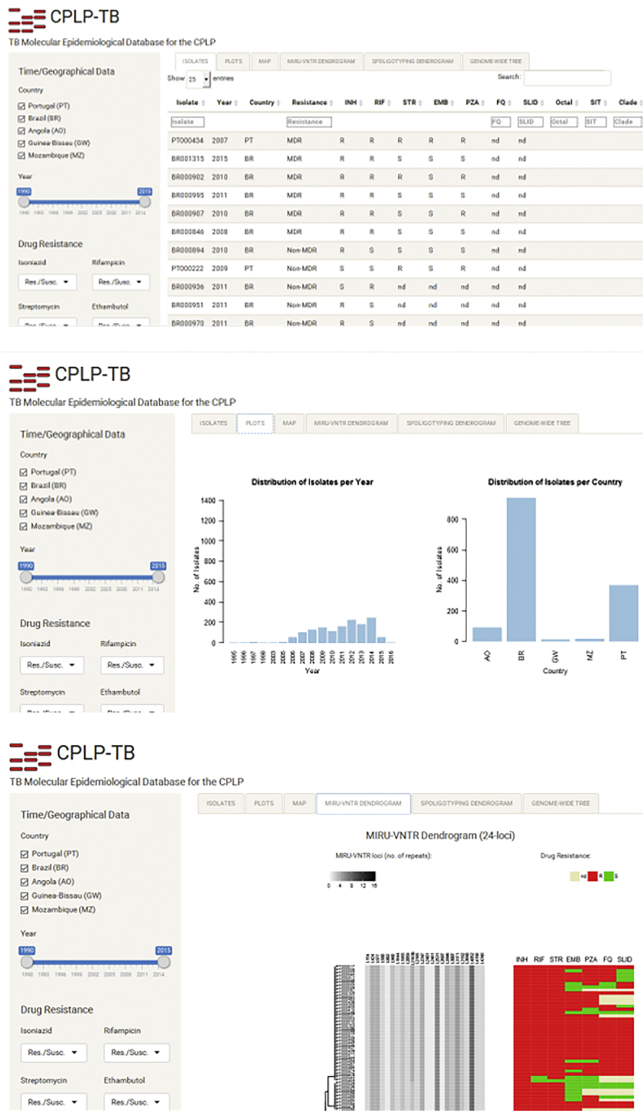
CPLP-TB database, an online tool purposely designed to house data on *M. tuberculosis* clinical isolates' originary from the CPLP, therefore enabling the tracking of specific strains across the Lusophone space. The database features a web interface composed of a main panel with six different page tabs and a left-sided panel that enables users to apply filters to data displayed on the main panel. Data displayed on the main panel include a data-table with strain information such as drug resistance, MIRU-VNTR alleles; different data plots showing enable analysis of the selected data: a choropleth map, which enables users to visualize the distribution of clinical isolates; MIRU-VNTR and Spoligotyping dendrograms; and, a genome-wide phylogenetic tree containing the isolates already subjected to WGS and annotated with the SNP barcode nomenclature.

To facilitate the dissemination and inter-laboratory comparison of the genotypic data generated in the present study we have created CPLP-TB, an online database and web application (available at http://cplp-tb.ff.ulisboa.pt/) (Fig. 5).

This database will accommodate more data on existing strains and, holds the potential to also accommodate more records on new strains and/or additional genotypic markers (such as SNPs or MIRU-VNTR hypervariable *loci*). Laboratories across the CPLP can also deposit data from additional settings and/or strains thus improving the comprehensiveness of this new database. With this database and web application it is also possible to track specific *M. tuberculosis* clones or monitor the *M. tuberculosis* population structure of a given country.

The main goal of this new tool is to provide a more comprehensive and global view of TB epidemiology in the CPLP. It holds several advantages in comparison to existing TB databases, which are intimately related with its main mission. CPLP-TB is intended to undergo through more rapid releases and continuous updates, including the addition of strains from underrepresented settings, that will enable to perform or contribute to risk assessment on specific strains at a more early stage; submission of data will be open to other laboratories provided that the strains deposited have been isolated within a CPLP country; it aggregates phenotypic and diverse genotypic data/markers and with the current architecture the database can be easily updated to accommodate additional epidemiological markers; it has been developed specifically for CPLP countries through CPLP-based laboratories, stimulating capacity development by local laboratories; and, CPLP-TB is linked to a biobank distributed across its hosting institution and its associated laboratories, thus enabling fundamental as well as applied research using clinically relevant *M. tuberculosis* strains.

### 3.8 Concluding remarks

The present study constitutes the first and largest collaborative effort to catalogue the *M. tuberculosis* diversity within the Lusophone space. A total of 1447 clinical isolates with origin in five countries, including 423 MDR-TB isolates, were analysed to provide a more global perspective of TB in the CPLP. The population structure covered by this analysis corroborates the high prevalence of LAM strains in most settings and its dispersion argues in favour of common populational structures, with past epidemiological links, driven by the radiation of this specific sub-lineage out of its setting of origin, which is presently unknown (Mokrousov et al., 2016).

Overall, this scenario argues in favour of the importance of global TB surveillance and it is in this context that CPLP-TB database can have a pivotal role in understanding shared transmission networks. In this regard, 24-loci MIRU-VNTR was found to be particularly useful and superior to the 15-loci set in analysing such a diverse dataset of clinical isolates, as it enabled the discrimination of 15-loci MIRU-VNTR clusters, which was of particular importance among cross-border clusters. Specifically, SIT20/LAM1 strains belonging to a newly identified cluster – CPLP-01 – emerge as the most important strains associated with more recent transnational strain flow. Most of the strains from this cluster were identified in Angola, which, given the present epidemiological scenario, makes it the likely origin of this particular cluster. Nonetheless, despite known limitations of both spoligotyping and MIRU-VNTRs, these typing methods remain the most used, easily-available, robust, and highly affordable methods – which makes them a method of choice for TB molecular epidemiology studies, particularly for developing countries. WGS based genomic epidemiology is not yet widely used in such settings. Future endeavours might consist in building new generation of databases able to establish useful links between data generated by “newer” (WGS) and “older” (spoligotyping/MIRU-VNTRs) molecular typing methods. Such a promising development includes a new tool named “SpoTyping” which allows in silico determination of *M. tuberculosis* spoligotyping patterns with a high accuracy from NGS reads, and subsequent lineage determinations by interrogating the SITVIT resources (Xia et al., 2016). Such developments will allow that the vast data generated using older genotyping methods are not denigrated and are instead useful to highlight the distribution summaries of the meta-data corresponding to *M. tuberculosis* lineages available in CPLP-TB and SITVIT databases. As such, this new tool, along with the several analysis and visualization tools, holds the potential for the analysis of TB transmission in these settings with an unprecedent resolution and can thus comprise a new framework for the analysis of *M. tuberculosis* genetic diversity within the Lusophone context and programmatic TB control.

## Funding

Financial support was provided by the European Society of Clinical Microbiology and Infectious Diseases, for which we would like to would like to acknowledge the Study Group for Mycobacterial Infections; Fundação CAPES [PVE-CAPES. 88881.064961/2014-01- Jose R. Lapa e Silva/UFRJ coordinator]; Genotyping and susceptibility profile of *Mycobacterium tuberculosis* clinical isolates from Rio Grande, Brazil were funded by Apoio a Projetos de Pesquisa em Doenças Negligenciadas, Brazil/MCTI/CNPq/MS-SCTIE – Decit [404081/2012-6] and by Programa Pesquisa para o SUS – PPSUS - FAPERGS/MS/CNPq/SESRS [1193-2551/13-6]; MIRU-VNTR typing and spoligotyping of *Mycobacterium tuberculosis* clinical isolates from Porto Alegre, Brazil were funded by National Council of Research [CNPq/MCTI/Universal - Project number: 441499/2014-7]; Fundação para a Ciência e a Tecnologia (FCT) Portugal [PTDC/SAU-EPI/122400/2010], part of the EDCTP2 program supported by the European Union; Fundação Calouste Gulbenkian, Portugal [Project ref. P-99934]. JP was supported by a post doc fellowship from project [PTDC/SAU-EPI/122400/2010] and by fellowship [SFRH/BPD/95406/2013] from FCT. The phylogenetic analysis work at Nalin Rastogi's lab was supported by a FEDER grant financed by the European Union and Guadeloupe Region (Programme Opérationnel FEDER-Guadeloupe-Conseil Régional 2014-2020, Grant number 2015-FED-192). IM was supported by Russian Science Foundation (grant 14-14-00292). AP was supported by a faculty baseline funding from KAUST [BAS/1/1020-01-01]. DM was supported by FCT fellowship [SFRH/BPD/100688/2014] and DM, IC MV are thankful to [GHTM UID/Multi/04413/20139] from FCT and to projects “ForDILAB-TB” and “A implementação de um novo método de identificação rápida do complexo M. tuberculosis nos Laboratórios de Referência da Tuberculose de Maputo e Beira” from Fundação Calouste Gulbenkian and the Community of the Portuguese Speaking Countries (CPLP). CS was supported by FCT [SFRH/BD/73579/2010]. TC is funded by the Medical Research Council UK (Grant no. MR/K000551/1 and MR/M01360X/1, MR/N010469/1, MC_PC_15103).

## Author contributions

JP, MV and IP designed and directed the study. JP, JD, ED, NT, SC, DM coordinated sample and data collection. JP, CS, JD, CP, DM, JR, HS, FA, CB, AR, MM, JS, AS, LE, RM, FM, SC, EC, SV, PR, AR, LJ, IC, AG, ED, MR, PS, MV and IP undertook sample collection, DNA extraction, genotyping and phenotypic drug susceptibility testing. JP, DC, NR and IM analysed the data and performed bioinformatic and statistical analyses. DC and NR performed comparative analysis against SITVIT. JP, AK, JLS, IM, DC, NR, ED, MR, PS, MV and IP interpreted results. JP wrote the ode of the online database. JP and IP wrote the first draft of the manuscript. TC, AP and RM generated preliminary whole-genome sequence data available online. JP, AK, JLS, NT, IM, DC, NR, ED, MR PS, MV and IP commented and edited on various versions of the draft manuscript and all authors approved the manuscript. JP, MV and IP compiled the final manuscript.

## Additional information

The authors have no competing financial interests to declare.

## Appendix A Supplementary data

Supplementary Figure S1 Image 19

Supplementary Figure S2 Image 20

Supplementary Figure S3 Image 21

Supplementary Figure S4 Image 22

Supplementary Table S1 Image 23

Supplementary Table S2 Image 24

Supplementary Table S3 Image 25

Supplementary Table S4 Image 26
