# Supplementary material for: Clonal expansion across the seas as seen through CPLP-TB database: A joint effort in cataloguing Mycobacterium tuberculosis genetic diversity in Portuguese-speaking countries
Source: Infect Genet Evol. 2019 Aug;72:44–58. doi: 10.1016/j.meegid.2018.03.011 (PMC6598853; doi:10.1016/j.meegid.2018.03.011)
Supplement: Supplementary file 1 — Supplementary Figure S1 [file mmc1.pdf]

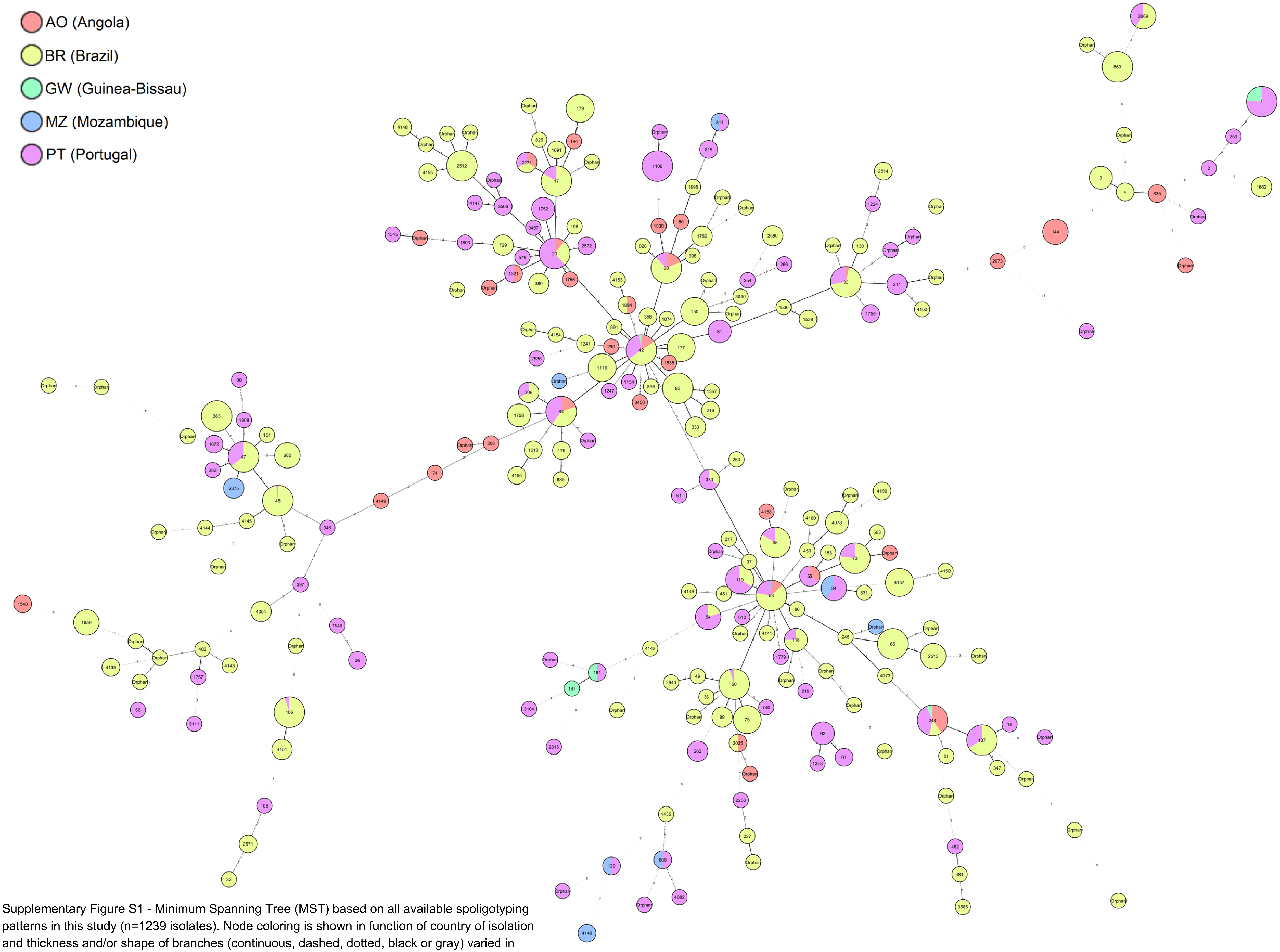

Supplementary Figure S1 - Minimum Spanning Tree (MST) based on all available spoligotyping patterns in this study (n=1239 isolates). Node coloring is shown in function of country of isolation and thickness and/or shape of branches (continuous, dashed, dotted, black or gray) varied in function of spacer changes between patterns. The number of changes is indicated on branches.
