# Supplementary material for: Clonal expansion across the seas as seen through CPLP-TB database: A joint effort in cataloguing Mycobacterium tuberculosis genetic diversity in Portuguese-speaking countries
Source: Infect Genet Evol. 2019 Aug;72:44–58. doi: 10.1016/j.meegid.2018.03.011 (PMC6598853; doi:10.1016/j.meegid.2018.03.011)
Supplement: Supplementary file 2 — Supplementary Figure S2 [file mmc2.pdf]

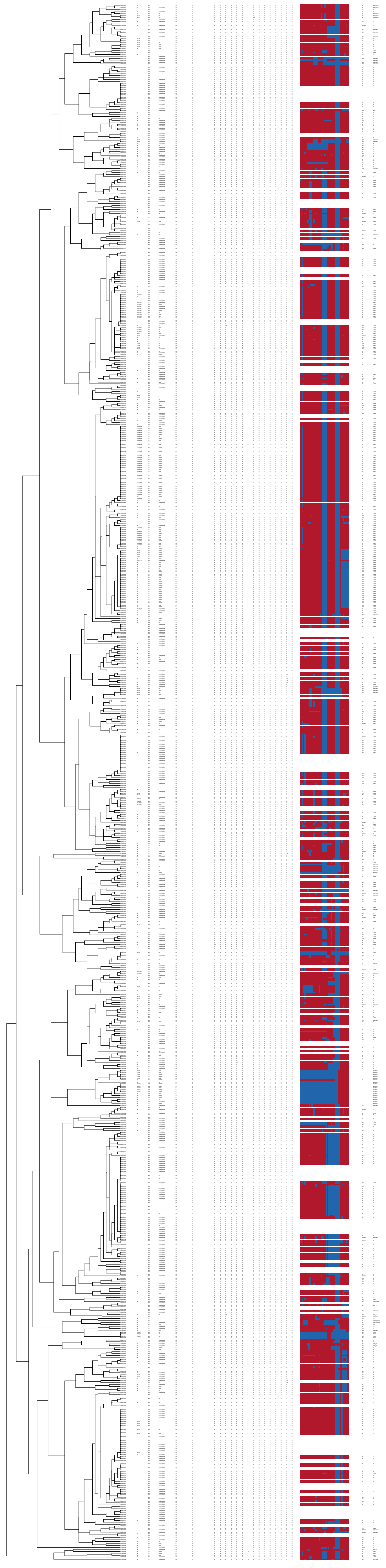

Supplementary Figure S2 – MIRU-VNTR dendrogram (15 loci) of 802 M. tuberculosis clinical isolates. Annotation: cluster identification, country of origin (ISO 3166), drug susceptibility data, MIRU-VNTR alleles, spoligotyping hybridization pattern, SIT and clade. First-line drug susceptibility testing: I, INH; R, RIF; S, STP; E, EMB; P, PZA. Second-line drug susceptibility testing: KAN, kanamycin; AMK, amikacin; CAP, capreomycin; OFX, ofloxacin; MOX, moxifloxacin; ETH, ethionamide; PAS, para-amino salicylic acid; CS, cycloserine; na, not available, nd, not determined.
