# Supplementary material for: Clonal expansion across the seas as seen through CPLP-TB database: A joint effort in cataloguing Mycobacterium tuberculosis genetic diversity in Portuguese-speaking countries
Source: Infect Genet Evol. 2019 Aug;72:44–58. doi: 10.1016/j.meegid.2018.03.011 (PMC6598853; doi:10.1016/j.meegid.2018.03.011)
Supplement: Supplementary file 5 — Supplementary Table S1 [file mmc5.pdf]

Supplementary Table S1: Spoligotype International Types (SIT) recorded in this study.

SIT	Spoligotype Description	Octal code	Nb in study	% in study	% in study vs. SITVIT	Lineage	Country Distribution**
1	□□□□□□□□□□□□□□□□□□□□□□□□□□□□□□□□□□■■■■■■■■■	000000000003771	33	2.66	0.31	Beijing	
2	□□□□□□□□□□□□□□□□□□□□□□□□■□□□□□□■□□□□■■■■■■■	000000004020771	1	0.08	0.2	H2	
3	□□□□□□□□□□□□□□□□□□□□□□□□■■■■■■□■□□□□■■■■■■■	000000007720771	4	0.32	3.64	H3	
4	□□□□□□□□□□□□□□□□□□□□□□□□■■■■■■■■□□□□■■■■■■■	000000007760771	2	0.16	0.49	Unknown	
17	■■□■■■■■■■■■□■■■■■■■□□□□■■■■■■■■□□□□■■■■■■■	677737607760771	42	3.39	5.62	LAM2	
18	■■□■■■■■■■■■■■■■■□■■■■■■■■■■■■■■□□□□■■□□□□■	677776777760601	1	0.08	2.44	X2	
20	■■□■■■■■■■■■■■■■■■■□□□□■■■■■■■■□□□□■■■■■■■	677777607760771	157	12.67	16.86	LAM1	
26	■■■□□□□■■■■■■■■■■■■■■■□□□□□□□□□□□□■■■■■■■■■	703777740003771	2	0.16	0.11	CAS1-Delhi	
32	■■■■■■■■□□□□□□□□□□□□□□□□□□□□□□□□□□□□■■■■■	776000000000171	1	0.08	0.97	Unknown	
33	■■■■■■■■□□□■■■■■■■■■□□□□■■■■■■■■□□□□■■■■■■■	776177607760771	29	2.34	1.99	LAM3	
34	■■■■■■■■□□■■■■■■■■■■■■■■■■■■■■■■□□□□■■■■■■■	776377777760771	5	0.4	0.56	S	
36	■■■■■■■■■■■■□■■■■■■■■■■■■■■■■■□■□□□□■■■■■■■	777737777720771	1	0.08	0.64	H3	
37	■■■■■■■■■■■■□■■■■■■■■■■■■■■■■■■■□□□□■■■■■■■	777737777760771	1	0.08	0.18	T3	
42	■■■■■■■■■■■■■■■■■■■■□□□□■■■■■■■■□□□□■■■■■■■	777777607760771	94	7.59	2.51	LAM9	
45	■■■■■■■■■■■■■■■■■■■■■■■□■□□□□□□■□□□□■■■■■■■	777777764020771	58	4.68	53.21	H1	
47	■■■■■■■■■■■■■■■■■■■■■■■■■□□□□□□■□□□□■■■■■■■	777777774020771	17	1.37	1.0	H1	
49	■■■■■■■■■■■■■■■■■■■■■■■■■■■■■■□■□□□□■■■□■■■	777777777720731	1	0.08	0.39	H3	
50	■■■■■■■■■■■■■■■■■■■■■■■■■■■■■■□■□□□□■■■■■■■	777777777720771	59	4.76	1.41	H3	
51	■■■■■■■■■■■■■■■■■■■■■■■■■■■■■■■■□□□□■■■□□□□	777777777760700	1	0.08	0.31	T1	
52	■■■■■■■■■■■■■■■■■■■■■■■■■■■■■■■■□□□□■■■□■■■	777777777760731	3	0.24	0.31	T2	
53	■■■■■■■■■■■■■■■■■■■■■■■■■■■■■■■■□□□□■■■■■■■	777777777760771	92	7.43	1.32	T1	
54	■■■■■■■■■■■■■■■■■■■■■■■■■■■■■■■■□□■■■■■■■■■	777777777763771	5	0.4	1.79	Manu2	
56	■■■■■■■■■■■■□■■■■■■■■■■□□□□□□□□□□□□□□□□□□□	777737770000000	1	0.08	3.23	Unknown	
58	■■■■■■■■■■■■■■■■■■■□■■□■■■■■■■■■■□□□□■■■■■■■	777777557760771	12	0.97	5.61	T5-Madrid2	
60	■■■■■■■■■■■■■■■■■■■■□□□□■■■■■■■■□□□□■■■□■■■	777777607760731	16	1.29	3.47	LAM4	
61	■■■■■■■■■■■■■■■■■■■■■■□□□■■■■■■■□□□□■■■■■■■	777777743760771	1	0.08	0.1	Cameroon	
64	■■■■■■■■■■■■■■■■■■■■□□□□■■■■□■■■□□□□■■■■■■■	777777607560771	15	1.21	3.26	LAM6	
65	■■■■■■■■■■■■■■■■■■■■■■■■■■■■■■■■□□□□■□□■■■■	777777777760471	60	4.84	42.86	T1	
73	■■■■■■■■■■■■□■■■■■■■■■■■■■■■■■■■□□□□■■■□■■■	777737777760731	13	1.05	4.11	T	
74	■■■■■■■■■■■■■■■■■■■■□□□□□□□□□■■■□□□□■■■■■■■	777777600160771	1	0.08	6.25	Unknown	
75	■■■■■■■■■■■□■■■■■■■■■■■■■■■■■■□■□□□□■■■■■■■	777677777720771	6	0.48	6.98	H3	
81	■■■■■■□■■■■■■■■■■■■■□□□□■■■■■■■■□□□□■■■■■■■	773777607760771	4	0.32	10.53	LAM9	
86	■■■■■■■■■■■■■■■■■■■■■□■■■■■■■■■■□□□□■■■■■■■	777777737760771	1	0.08	1.03	T1	
90	■■□■■■■■■■■■■■■■■■■■■■■■■□□□□□□□□□□□■■■■■■	677777776000371	1	0.08	2.94	Unknown	
91	■■■□□□□□□□□□□■■■■□■■■■■■■■■■■■■■□□□□■■■■■■■	700036777760771	2	0.16	0.54	X3	
92	■■■□□□□□□□□□■■■■■□■■■■■■■■■■■■■■□□□□■■■■■■■	700076777760771	4	0.32	0.87	X3	
93	■■■■■■■■■■■■□■■■■■■■□□□□■■■■■■■■□□□□■■■■■■■	777737607760771	28	2.26	5.87	LAM5	
95	■■■■■■■■■■■■■■■■■■■■□□□□■■■■□■■■□□□□■■■□■■■	777777607560731	1	0.08	1.16	LAM6	
99	■■■■□■■■■■■■■■■■■■■■■■■■■■■■■■□■□□□□■■■■■■■	757777777720771	3	0.24	3.66	H3	
105	■■■■■■■■□□□■■■□□□□□□□□□□□□□□□□□□□□□□□□□■■■■	776160000000071	1	0.08	1.89	Unknown	
106	■■■■■■■■□□□■■■■■■■■□□□□□□□□□□□□□□□□□□□■■■■■	776177400000171	20	1.61	13.7	Unknown	
118	■■■■■■■■■■■■■■□■■■■■■■■■■■■■■■■■□□□□■■■■■■■	777767777760771	4	0.32	2.31	T1	
119	■■■■■■■■■■■■■■■■■□■■■■■■■■■■■■■■□□□□■■■■■■■	777776777760771	6	0.48	0.54	X1	
129	■■■□□□□□□■■■■■■■■■■■■■□□■■■■□□□□■□■■■■■■■■■	700777747413771	2	0.16	3.08	EAI6-BGD1	
130	■■■■■■■■□□□■■■■■■■■■□□□□■■■■■■■■□□□□■■■□■■■	776177607760731	1	0.08	0.72	LAM3	
137	■■■■■■■■■■■■■■■■■□■■■■■■■■■■■■■■□□□□■■□□□□■	777776777760601	12	0.97	1.12	X2	
144	■■■■■■□□□□□□□□□□□□□□□□□□□■■■■■■■□□□□■■■■■■■	770000003760771	5	0.4	8.2	Unknown	
150	■■■■■■■■■■■■■■□■■■■■□□□□■■■■■■■■□□□□■■■■■■■	777767607760771	6	0.48	5.66	LAM9	
151	■■■■■■■■■■■■■■□■■■■■■■■■■□□□□□□■□□□□■■■■■■■	777767774020771	1	0.08	3.7	H1	
153	■■■■□■■■■■■■■■■■■■■■■■■■■■■■■■■■□□□□■■■□■■■	757777777760731	1	0.08	1.27	T2	
176	■■■■■■■■■■■■□■■■■■■■□□□□■■■■□■■■□□□□■■■■■■■	777737607560771	2	0.16	4.88	LAM6	
177	□■■■■■■■■■■■■■■■■■■■□□□□■■■■■■■■□□□□■■■■■■■	377777607760771	6	0.48	4.65	LAM9	
179	■■□■■■■■■■■■□■■■■■■■□□□□■■■■■■■■□□□□■■■□□■■	677737607760711	6	0.48	27.27	LAM	
181	■■■■■■□□□■■■■■■■■■■■■■■■■■■■■■■■■■■■□■■■■	770777777777671	2	0.16	0.69	AFRI_1	
187	■■■■■■□□□■■■■■■■■■■■■■■■■■■■■■■■□■■■■■□■■■■	770777777767671	1	0.08	1.01	AFRI_1	
194	■■□■■■■■■■■■□■■■■■■■□□□□■■■■■■■■□□□□■■■□■■■	677737607760731	1	0.08	3.03	LAM2	
195	■■□■■■■■■■■■■■■■■■■■□□□□■■■■■□■■□□□□■■■■■■■	677777607660771	1	0.08	6.25	LAM1	
211	■■■■■■■■□□□■□■■■■■■■□□□□■■■■■■■■□□□□■■■■■■■	776137607760771	3	0.24	2.52	LAM3	
216	■■■■■■■■■■■■□□■■■■■■□□□□■■■■■■■■□□□□■■■■■■■	777717607760771	2	0.16	3.64	LAM5	
217	■■■■■■■■■■■■□■■■■□■■■■■■■■■■■■■■□□□□■■■■■■■	777736777760771	1	0.08	2.27	X1	
219	■■■■■■■■■■■■■□□□□□■■■■■■■■■■■■■■□□□□■■■■■■■	777740777760771	1	0.08	1.16	T1	
237	■■■■■■■■■■■■■■■■■■■■■■■■■■■■■■□□□□□□□□□□□□□	777777777700000	1	0.08	0.76	Unknown	
244	■■■■■■■■■■■■■■■■■■■■■■■■■■■■■■■■□□□□■■□□□□■	777777777760601	15	1.21	10.64	T1	
245	■■■■■■■■■■■■■■■■■■■■■■■■■■■■■■■■□□□□■■□■■■■	777777777760671	1	0.08	4.35	T1	
250	□□□□□□□□□□□□□□□□□□□□□□□□□□□□□□□□□□□□□■■■■■■	000000000000371	1	0.08	2.56	Beijing	
253	■■■■■■■■■■■■■■■■■■■■□■■□□■■■■■■■□□□□■■■■■■■	777777663760771	1	0.08	1.82	T1	
254	■■■■■■■■■■■■■■□□□□□□□□□□■■■■■■■■□□□□■■■■■■■	777760007760771	1	0.08	0.53	T5-RUS1	
262	■■■■■■■□□■■■■■■■■■■■■■■■■■■■□□□■□□□□■■■■■■■	774777777420771	3	0.24	1.4	Ural-1	
266	■■■■■■■□■■■■■□□□□□□□□□□□□■■■■■■■□□□□■■■■■■■	775740003760771	1	0.08	6.67	T1	
290	■■■■■■■■■■■■■■■■■■■■□□□□■■□■■■■■□□□□■■■■■■■	777777606760771	1	0.08	3.23	LAM8	
306	■■■■■■■■■■■■■■■■■■■■□□□□□□■■□■■■□□□□■■■■■■■	777777601560771	1	0.08	3.33	T1	
333	■■■■■■■■■■■■□■□■■■■■□□□□■■■■■■■■□□□□■■■■■■■	777727607760771	3	0.24	25.0	LAM5	
347	■■■■■■■■■■■■□■■■■□■■■■■■■■■■■■■■□□□□■■□□□□■	777736777760601	1	0.08	1.92	X2	
373	■■■■■■■■■■■■■■■■■■■■■■■□■■■■■■■■□□□□■■■■■■■	777777767760771	3	0.24	4.23	T1	
382	■■■■□■■■■■■■■■■■■■■■■■■■■□□□□□□■□□□□■■■■■■■	757777774020771	1	0.08	7.69	H1	
383	■■■■■■■■□□■■■■■■■■■■■■■■■□□□□□□■□□□□■■■■■■■	776377774020771	8	0.65	33.33	H1	

388	■■■□■■■■■■■■■■■■■■■■□□□□■■■■■■■■□□□□■■■■■■■	737777607760771	2	0.16	6.45	LAM9		
389	■■□■■■■■■■■■■■□■■■■■□□□□■■■■■■■■□□□□■■■■■■■	677767607760771	3	0.24	15.0	LAM1		
396	■■■■■■■■■■■■■■■■■■■■□□□□■■■■□■■■□□□□□■■■■■■	777777607560371	3	0.24	15.79	LAM6		
397	■■■■■■■■■■■■■■■■■■■■□□□□□□□□□□□□□□□□■■■■■■■	777777600000771	1	0.08	3.85	LAM		
398	■■■■■■■■■■■■■■■■■■■■□□□□■■■■■■■■□□□□■■□□■■■	777777607760631	1	0.08	5.0	LAM4		
402	■■■■■■■■■■■■■■■■■■■■□□□□□□□□□□□□□□□□□□□□□□□	777777600000000	1	0.08	2.22	Unknown		
451	■■■■■■■■■■■■■■■■■■■□□■■■■■■■■■■■□□□□■■■■■■■	777777477760771	1	0.08	0.68	T-H37Rv		
453	■■■■■■■■■■■■■■■■■■■■□■■■■■■■□■■■□□□□■■■■■■■	777777677560771	1	0.08	4.76	T1		
481	■■□■■■■■□■■■■■■□■■■■□■■■■■■■■■■■■■■■■■□□□□□	676773677777600	1	0.08	0.02	BOV_1		
482	■■□■■■■■□■■■■■■□■■■■■■■■■■■■■■■■■■■■■■□□□□□	676773777777600	1	0.08	0.06	BOV_1		
503	■■■■■■■■■■■■□■■■■■□■■■■■■■■■■■■■□□□□■■■□■■■	777737377760731	1	0.08	25.0	T		
578	■■□□■■■■■■■■■■■■■■■■□□□□■■■■■■■■□□□□■■■■■■■	637777607760771	1	0.08	2.78	LAM1		
602	■■■■■■■■■■■■■■■■■■■■■■■■□□□□□□□□□□□□■■■■■■■	777777770000771	5	0.4	4.81	Unknown		
612	■■■■■■■■■■■■■■■■■■■■■■■■■■■■■■■■□□□□■■■■□■■	777777777760751	1	0.08	2.94	T1		
635	□□□□□□□□□□□□□□□□□□□□□□□□■■■■□■■■□□□□■■■■■■■	000000007560771	2	0.16	16.67	LAM3		
729	■■□■□■■■■■■■■■■■■■■■□□□□■■■■■■■■□□□□■■■■■■■	657777607760771	3	0.24	23.08	LAM1		
740	■■■■■■■■■■■■■■■■■■■■■■□□■■■■■■□■□□□□■■■■■■■	777777747720771	1	0.08	3.85	H3		
806	■■■■□■■■■■■■■■■■■■■■■■■■■■■■□□□□■□■■■■■□■■■	757777777413731	2	0.16	3.7	EAI1-SOM		
811	■■■■■■■■■■■■■■■■■■■■□□□□■□□□□□■■□□□□■■■□■■■	777777604060731	2	0.16	3.51	LAM11-ZWE		
815	■■■■■■■■■■■■■■■■■■■■□□□□■■□□□□■■□□□□■■■□■■■	777777606060731	2	0.16	1.34	LAM11-ZWE		
826	■■□■■■■■■■■■□■■■■■□■□□□□■■■■■■■■□□□□■■■■■■■	677737207760771	1	0.08	7.14	LAM2		
828	□■■■■■■■■■■■■■■■■■■■□□□□■■■■■■■■□□□□■■■□■■■	377777607760731	1	0.08	2.86	LAM4		
831	■■■■■■■■□□■■■■□■■■■■■■■■■■■■■■■■□□□□■■■■■■■	776367777760771	1	0.08	7.14	S		
863	□□□□□□□□□□□□□□□□□□□□□□□□■■■■□□□□□□□□□□□□□□□	000000007400000	28	2.26	560.0	PINI2		
866	■□■■■■■■■■■■■■■■■■■■□□□□■■■■■■■■□□□□■■■■■■■	577777607760771	1	0.08	1.69	LAM9		
885	■■■■■■■■■■■■□■■■■■■■□□□□■■■■□□■■□□□□■■■■■■■	777737607460771	1	0.08	50.0	LAM5		
891	■■■■■■■■■■■■■■■■■■■■□□□□■■■■■□■■□□□□■■■■■■■	777777607660771	1	0.08	3.33	LAM9		
946	■■■■■■■■■■■■■■■■■■■■■■□□□□□□□□□■□□□□■■■■■■■	777777740020771	1	0.08	4.76	H		
1074	■■■■■■■■■■■■■■■■■■■■□□□□■■■■■■■■□□□□■■■■■□■	777777607760761	1	0.08	3.13	LAM9		
1106	■■■■■■■■■■■■■■■■■■■■□□□□■■■■■■■■□□□□■□□□□□□	777777607760400	38	3.07	172.73	LAM4		
1154	■■■■■■■■■■■■■■■■■■■■□□□□■■■■■■■■□□□□■■■■□■■	777777607760751	1	0.08	5.26	LAM9		
1176	■■■■■■■■■■■■■□■■■■■■□□□□■■■■■■■■□□□□■■■■■■■	777757607760771	6	0.48	30.0	LAM9		
1224	■■■■■■■■□□□■■■■■■■■■□□□□■■■■■■■■□□□□■■■□□□□	776177607760700	1	0.08	12.5	LAM3		
1241	■■■■■■■■■■■■■■■■■■■■□□□□■■■■■■□□□□□□■■■■■■■	777777607700771	2	0.16	2.2	LAM9		
1247	■■■■■■■■■■■■■■■■■■■■□□□□■■■■■■■■□□■■■■■■■■■	777777607763771	1	0.08	6.67	Manu2		
1273	■■■□□□□□□□□□■■■■■□■■■■■■■■■■■■■■□□□□■■■□■■■	700076777760731	1	0.08	12.5	X3		
1321	■■□■■■■■■■■■■■■■■■■■□□□□■■■■■■■■□□□□■■■□■■■	677777607760731	2	0.16	6.9	LAM		
1367	□■■■■■■■■■□■■■■■■■■■□□□□■■■■■■■■□□□□■■■■■■■	377737607760771	1	0.08	16.67	LAM5		
1435	■■■■■■■□□■■■■■■■■■■■■■■■■■■■□□□□■□■■■■■□■■■	774777777413731	1	0.08	25.0	EAI1-SOM		
1528	■■■■■■■□□□■■■■■■■■■■□□□□■■■■■■■■□□□□■■■■■■■	774377607760771	2	0.16	66.67	LAM9		
1530	■■■■■■■■■■■■■■■■■■■■□□□□■■■■■■■■□□□□■■■□□■■	777777607760711	1	0.08	8.33	LAM4		
1535	■■■■■■■■■■□■■■■■■■■■□□□□■■■■■■■■□□□□■■■■■■■	777577607760771	1	0.08	7.14	LAM9		
1536	■■■■■■■■□□■■■■■■■■■■□□□□■■■■■■■■□□□□■■■■■■■	776377607760771	1	0.08	4.76	LAM9		
1545	■■□□□□□□■■■■■■■■□■■■□□□□■■■■■■■■□□□□■■■■■■■	601775607760771	1	0.08	16.67	LAM1		
1548	■■■■■■■■■■■■□□□□■■■□□□□□□□□□□□□□□□□□□□■■■■□□	777703400000360	2	0.16	25.0	Unknown		
1610	■■■■■□□■■■■■■■■■■■■■□□□□■■■■□■■■□□□□■■■■■■■	763777607560771	2	0.16	33.33	LAM6		
1659	■■■■■■■■■■■■□□□□■■■■■□□□□□□□□□□□□□□□□□□□□□	777703700000000	5	0.4	100.0	Unknown		
1662	■■□□□□□□□□□□□□□□□□□□□□□□□□□□□□■■□□□□■■■□■■■	600000000060731	3	0.24	150.0	T2		
1691	■■□■■■■■■■■■□■□■■■■■□□□□■■■■■■■■□□□□■■■■■■■	677727607760771	2	0.16	10.53	LAM2		
1750	■■■■■■■■■■■■■■□■■■■■□□□□■■■■■■■■□□□□■■■□■■■	777767607760731	3	0.24	33.33	LAM4		
1752	■■□■■■■■■■■■■■■■■■■■□□□□■■■■■■■□□□□□■■■■■■■	677777607740771	4	0.32	133.33	LAM1		
1755	■■□■■■■■■■■■■■■■■■■■□□□□■■■■□■■■□□□□■■■■■■■	677777607560771	1	0.08	10.0	LAM6		
1757	■■■■□■■■■■■■■■■■■■■■□□□□□□□□□□□□□□□□□□□□□□□	757777600000000	1	0.08	33.33	Unknown		
1758	■■■■■■■■■■■■■■■■■■■■□□□□■■■■□■■□□□□□■■■■■■■	777777607540771	4	0.32	33.33	LAM9		
1759	■■■■■■□■□□□■■■■■■■■■□□□□■■□■■■■■□□□□■■■■■■■	772177606760771	2	0.16	66.67	LAM3		
1779***	■■■■■■■■□■□□■■■■■■■■■■■■■■■■■■■■□□□□■■■■■■■	776477777760771	1	0.08	NA	Unknown		
1803	■■□□□■■■■■■■■■■■■■■■□□□□■■■■■■■■□□□□■■■■■■■	617777607760771	1	0.08	7.14	LAM1		
1872	■■■■■■■■■■■■■■■■■■■■■■■■■□□□□□■■□□□□■■■■■■■	777777774060771	2	0.16	40.0	T1		
1894	■■■■□□■■■■■■■■■■■■■■□□□□■■■■■■■■□□□□■■■■■■■	747777607760771	2	0.16	66.67	LAM9		
1895	■■■■■■■■■■■■■■■■■■■■□□□□■■■■□□■■□□□□■■■□■■■	777777607460731	1	0.08	6.67	LAM4		
1908	■■■■■■■■■■■■■■■■■■■■■■■■■■□□□□□■□□□□■■■■■■■	777777776020771	1	0.08	16.67	H3		
1949	■■■□□□□■■■■■■■■■■■■■■□□□□□□□□□□□□□□□■■■■■■■	703777700000771	1	0.08	5.88	CAS		
2025	■■■■■■■■■■■■■■■■■■■■■■■■■■■■■■□□□□□□□■■■■■■	777777777700371	2	0.16	16.67	T		
2073	■■■■■■■■□□□□□□□□□□■■□□□□■■■■■■■■□□□□■■■■■■■	776000607760771	1	0.08	25.0	LAM3		
2258	■■■■■■■■■■■■■■■■■■■■■■■■■■■■■■□□□□□□□□□□□■■	777777777700011	1	0.08	8.33	Unknown		
2271	■■□■■■■■■■■■□□■■■■■■□□□□■■■■■■■■□□□□■■■■■■■	677717607760771	3	0.24	30.0	LAM2		
2375	■■■■■□■■■■■■■■■■■■■■■■■■■■■■□□□□□□■□□□□■■■■■■■	767777774020771	3	0.24	21.43	H1		
2506	■■□■■■■■■■■■■■□■■■■■■□□□□■■■■■■■■□□□□■■■■■■■	677757607760771	2	0.16	40.0	LAM1		
2512	■■□■□■■■■■■■■□■■■■■■□□□□■■■■■■■■□□□□■■■■■■■	657757607760771	19	1.53	316.67	LAM1		
2513	■■■■■■■■■■■■■■□■■■■■■■■■■■■■■■■■□□□□■□□■■■■	777767777760471	5	0.4	125.0	T1		
2514	■■■■■■■□□□■■■■■■■■■■□□□□■■■■■■■■□□□□■■■□□□□	774377607760700	2	0.16	28.57	LAM4		
2535	■■■■■■■■■■■■■■■■■■□■□□□□■■■■■□□□□□□□□■■□■■■	777777207600331	1	0.08	5.88	Unknown		
2571	■■■■■■■■□□■■■□□□□□□□□□□□□□□□□□□□□□□□□□■■■■■	776340000000171	2	0.16	66.67	Unknown		
2572	■■□■■□■■■■■■■■■■■■■■□□□□■■■■■■■■□□□□■■■■■■■	667777607760771	2	0.16	50.0	LAM1		
2580	■■■■■■■■■■■■■□□□□□□□□□■■■□□■■■■□□□□■■■■■■■	777740016360771	3	0.24	150.0	T1		
2640	■■□■■■■■■■■■■■■■■■■■■■■■■■■■■■□■□□□□■■■□■■■	677777777720731	1	0.08	50.0	H3		
2669	□□□	000000000000000	5	0.4	4.55	ATYPIC		
2815	■■■■■■□□□■■■■■■■■■■□□□□□■□□□□■■■■■■■■■□■■■■	770777404177671	1	0.08	50.0	AFRI_1		
3040	■■■■■■■■■□■■■■□■■■■■□□□□■■■■■■■■□□□□■■□■■■■	777367607760671	1	0.08	50.0	LAM9		CO=1, ES=1
3104	■■■■■□□□□□■■■■■■■■■■■■■■■■■■■■■□□□■■■■□■■■■	760377777743671	1	0.08	50.0	AFRI_1		GW=2
3111	■■■□□□□■■■■■■■■■■■■■■□□□□□□□□□□□□□□□□□□□□□□	703777700000000	1	0.08	50.0	CAS_1-Delhi		GW=2

3450	■■■■■■■■■■■■■■■■■■■□□□□□■□■■■■□■□□□□■■■■■■■	777777405720771	1	0.08	33.33	H3	FI=1, ES=1, FR=1
3457	■■□■■■■□■■■■■■■■■■■■□□□□■■■■■■■■□□□□■■■■■■■	675777607760771	1	0.08	50.0	LAM1	FR=1, VE=1
3585	■□□■■■■■□■■■■■□□■■■■□■■■■■■■■■■■■■■■■■□□□□□	476763677777600	1	0.08	12.5	BOV	ES=8
4004	■■■■■■■■■■■■■■■■■■■■□□□□□□□□□□□□□□□□□■■□■■■	777777600000331	3	0.24	100.0	Unknown	BR=3
4073	■■■■■■■■■■■■■■■■■■■■■■■■■■■■■■■■□□□□■■□■■□■	777777777760661	1	0.08	50.0	T1	ZM=1, BR=1
4078	■■■■■■■■■■■■■■■■■■■■□■■■■■■■□■■■□□□□■■■□■■■	777777677560731	4	0.32	200.0	T2	BR=2
4092	■■■■■■■■■■■■■■■■■■■■■□□■■■■■□□□□■□■■■■■□■■■	777777717413731	1	0.08	50.0	EAI1-SOM	MZ=2
4139*	■■■■■■■■■■■■□■■■■■□■□□□□■■■■□□□□□□□□□□□□□□□	777737207400000	2	0.16	100.0	Unknown	this study n=2
4140*	■■■□□□□□□■■■■■■■■■■■■■□□■■■□□□□□□□□□□□□■■■■	700777747000071	2	0.16	100.0	Unknown	this study n=2
4141*	■■■■■■■■■■■■■■■■■■■■■■■■■■■■■■■■□■■□■■■■■■■	777777777766771	1	0.08	50.0	Unknown	this study n=1, NG=1
4142*	■■■■■■■■■■■■■■■■■■■■□■■■■■■■■■■■■■■■■□■■■■	777777677777671	1	0.08	50.0	Unknown	this study n=1, US=1
4143*	■■■■■■□□■■■■■■■■■■■■□□□□□□□□□□□□□□□□□□□□□□	771777600000000	1	0.08	50.0	Unknown	this study n=1, BR=1
4144*	■□■■□■■■■■■■■■□■■■■■■■■□■□□□□□□□□□□□■■■■■■■	557767764000771	1	0.08	50.0	Unknown	this study n=1, BR=1
4145*	■■■■■■■■■■■■■■□■■■■■■■■□■□□□□□□□■□□□□■■■■■■■	777767764020771	1	0.08	50.0	H1	this study n=1, DE=1
4146*	■□■■■■■■■■■■■■□■■■■□□■■■■■■■■■■■□□□□■■■■■■■	577767477760771	1	0.08	50.0	T-H37Rv	this study n=1, GB=1
4147*	■■□■■■■■■■■■■□□■■■■■□□□□■■■■■■■■□□□□■■■■■■■	677747607760771	1	0.08	50.0	LAM1	this study n=1, AR=1
4148*	■□□■□■■■■■■■■□□■■■■■□□□□■■■■■■■■□□□□■■■■■■■	457747607760771	2	0.16	100.0	LAM1	this study n=2
4149*	■■■■■■■■■■■■■■■■■■■■■■□□□□□□□■■■□□□□■■■■■■■	777777740160771	1	0.08	50.0	T1	this study n=1, AT=1
4150*	■■■■■■■■□□■■□■■■■■■■■□□□□□□■■■■■□□□□■■■■■■■	776337700760771	1	0.08	50.0	S	this study n=1, BR=1
4151*	■■■■■■■■□□□■■■□■■■■■□□□□□□□□□□□□□□□□□□■■■■■	776167400000171	3	0.24	75.0	Unknown	this study n=3, ES=1
4152*	■■■■■■■■■□□□□■■■■■■■■□□□□■■■■■■■■□□□□■■■■■■■	777037607760771	1	0.08	50.0	LAM5	this study n=1, BR=1
4153*	■■■■□□■■■■■■■■■■■■■■□□□□■■■■□■■■□□□□■■■■■■■	747777607560771	1	0.08	50.0	LAM6	this study n=1, BR=1
4154*	■■■■■■■■□■■■■■■■■■■■□□□□■■■■■■□□□□□□■■■■■■■	776777607700771	1	0.08	50.0	Unknown	this study n=1, BR=1
4155*	■■■■□■■■■■■■■□■■■■■■□□□□■■■■■■■■□□□□■■■■■■■	757757607760771	2	0.16	100.0	LAM9	this study n=2
4156*	■■■■■□□■■■■■■■□■■■■■□□□□■■■■□■■■□□□□■■■■■■■	763767607560771	2	0.16	100.0	LAM6	this study n=2
4157*	■■■■■■■■□□■■■■■■■■■■■■■□□□□■■■■■□□□□■■■■■■■	776377760760771	6	0.48	100.0	S	this study n=6
4158*	■■■■■■■■■■■■■■■■■■■□■■□■■■■■□■■■□□□□■■■■■■■	777777557560771	1	0.08	50.0	T1	this study n=1, AR=1
4159*	■■■■■■■■■■■■■■■■■■■■□■■■□□□□□■■■□□□□■■■□■■■	777777670160731	2	0.16	100.0	T1	this study n=2
4160*	■■■■■□□■■■■■■■■■■■■■□■■■■■■■□■■■□□□□■■■■■■■	763777677560771	1	0.08	50.0	T1	this study n=1, BR=1

Newly created SITs are followed by an asterisk (*)

** Country distribution was provided for recently created SITs (the 2 letter country codes are according to http://en.wikipedia.org/wiki/ISO_3166-1_alpha-2)

*** SIT1779 in SITVITWEB was deleted in SITVIT2 database; the pattern is however retained pending its reassignement if new strains are reported in future.
