# Supplementary material for: Clonal expansion across the seas as seen through CPLP-TB database: A joint effort in cataloguing Mycobacterium tuberculosis genetic diversity in Portuguese-speaking countries
Source: Infect Genet Evol. 2019 Aug;72:44–58. doi: 10.1016/j.meegid.2018.03.011 (PMC6598853; doi:10.1016/j.meegid.2018.03.011)
Supplement: Supplementary file 6 — Supplementary Table S2 [file mmc6.pdf]

Supplementary Table S2: Description of clusters containing 12 or more isolates in this study, and their worldwide distribution in the SITVIT database.

SIT	Spoligotype Description	Octal code	Nb in study	% in study	% in study vs. database	Lineage	Distribution in Countries with ≥2% of a given SIT*	Distribution in Regions with ≥2% of a given SIT**
1	□□□□□□□□□□□□□□□□□□□□□□□□□□□□□□□□□□■■■■■■■■■	000000000003771	33	2.66	0.31	Beijing	CN=19.12, US=18.89, JP=10.86, ZA=7.81, RU=6.6, VN=3.67, IN=3.56, PE=2.96, MY=2.79	Eastern Asia=32.19, North America=18.93, Southeastern Asia=10.06, Southern Africa=7.81, Northern Asia=6.6, Southern Asia=5.92, Northern Europe=3.45, South America=3.42, Western Asia=2.81, Western Europe=2.42
17	■■□■■■■■■■■■□■■■■■■■□□□□■■■■■■■■□□□□■■■■■■■	677737607760771	42	3.39	5.62	LAM2	BR=30.52, VE=23.96, US=15.93, HT=7.36, ES=3.88, GP=2.68, CO=2.41	South America=59.97, North America=15.93, Caribbean=11.91, Southern Europe=4.95, Southern Africa=2.01
20	■■□■■■■■■■■■■■■■■■■■□□□□■■■■■■■■□□□□■■■■■■■	677777607760771	157	12.67	16.86	LAM1	BR=17.62, US=17.08, HT=7.3, NA=6.66, PT=5.26, FR=5.26, VE=4.51, ZA=2.9, ES=2.69, AR=2.36, PE=2.26, CO=2.15, BE=2.04	South America=31.36, North America=17.08, Western Europe=9.88, Southern Africa=9.56, Caribbean=9.13, Southern Europe=8.49, East Africa=3.97, Northern Europe=2.79, West Africa=2.69
33	■■■■■■■■□□□■■■■■■■■■□□□□■■■■■■■■□□□□■■■■■■■	776177607760771	29	2.34	1.99	LAM3	ZA=22.26, PE=14.38, BR=11.1, US=10.89, AR=10.62, ES=6.1, CL=3.77, FR=3.7, IT=3.15, HN=2.95	South America=41.58, Southern Africa=22.26, North America=10.89, Southern Europe=9.73, Western Europe=5.62, Central America=3.7, North Africa=2.19
42	■■■■■■■■■■■■■■■■■■■■□□□□■■■■■■■■□□□□■■■■■■■	777777607760771	94	7.59	2.51	LAM9	BR=14.45, US=10.6, CO=7.64, MA=6.3, IT=5.85, FR=4.54, AR=3.53, PE=3.31, ES=2.99, VE=2.96, ZA=2.83, HT=2.16, RU=2.06	South America=35.02, North America=10.6, Southern Europe=10.12, Western Europe=8.47, North Africa=7.67, Northern Europe=4.38, Caribbean=3.79, East Africa=3.47, Central America=3.18, Southern Africa=2.83, Northern Asia=2.06
45	■■■■■■■■■■■■■■■■■■■■■■■□■□□□□□□■□□□□■■■■■■■	777777764020771	58	4.68	53.21	H1	AT=16.51, MQ=11.01, US=9.17, CO=8.26, IT=8.26, BR=8.26, GP=7.34, DE=3.67, NL=2.75, LC=2.75, SE=2.75	Western Europe=23.85, Caribbean=22.02, South America=19.27, Southern Europe=11.01, North America=9.17, Northern Europe=6.42
47	■■■■■■■■■■■■■■■■■■■■■■■■■□□□□□□■□□□□■■■■■■■	777777774020771	17	1.37	1.0	H1	US=13.96, IT=7.57, AT=7.39, BR=7.16, PE=6.92, FR=6.1, FI=5.51, CZ=3.46, ES=3.28, SE=3.11, AR=2.64, MA=2.58, NL=2.29, CF=2.17, TR=2.05	South America=18.71, Western Europe=18.65, North America=14.25, Southern Europe=12.38, Northern Europe=9.62, Eastern Europe=6.63, Western Asia=3.7, North Africa=3.34, Caribbean=2.52, Central Africa=2.4
50	■■■■■■■■■■■■■■■■■■■■■■■■■■■■■■□■□□□□■■■■■■■	777777777720771	59	4.76	1.41	H3	US=14.2, PE=12.6, BR=7.85, FR=5.56, AT=4.92, ES=4.39, IT=4.39, ZA=3.27, AR=3.08, CM=3.01, CZ=2.96, HT=2.77, TR=2.58, SE=2.29	South America=28.77, Western Europe=14.2, North America=14.2, Southern Europe=9.33, Caribbean=4.7, Eastern Europe=4.46, Northern Europe=4.42, Western Asia=3.63, North Africa=3.44, Southern Africa=3.27, Central Africa=3.06, Eastern Asia=2.03
53	■■■■■■■■■■■■■■■■■■■■■■■■■■■■■■■■□□□□■■■■■■■	777777777760771	92	7.43	1.32	T1	US=11.96, FR=7.14, BR=5.87, IT=4.82, TR=4.51, ZA=4.39, PE=4.29, AT=3.09, CN=2.82, FI=2.65, MX=2.58, ET=2.35, SA=2.26, AR=2.19, ES=2.09	South America=17.09, Western Europe=14.15, North America=12.19, Southern Europe=8.51, Western Asia=7.97, Northern Europe=6.77, East Africa=4.65, Southern Africa=4.49, Eastern Asia=3.86, North Africa=3.18, Eastern Europe=2.95, Central America=2.92, Caribbean=2.88, Southern Asia=2.86
58	■■■■■■■■■■■■■■■■■■■□■■□■■■■■■■■■■□□□□■■■■■■■	777777557760771	12	0.97	5.61	T5-Madrid2	US=21.96, ES=19.63, PE=11.68, AR=11.68, BR=8.88, FR=6.07, MA=2.8, CL=2.34, CU=2.34, NL=1.87, VE=1.87, TN=1.4, BE=0.93, PT=0.93, IT=0.93, ZA=0.93, AT=0.93, PY=0.47, DZ=0.47, MQ=0.47, GF=0.47, SE=0.47, MX=0.47	South America=37.38, North America=21.96, Southern Europe=21.5, Western Europe=9.81, North Africa=4.67, Caribbean=2.8
60	■■■■■■■■■■■■■■■■■■■■□□□□■■■■■■■■□□□□■■■□■■■	777777607760731	16	1.29	3.47	LAM4	ZA=35.36, BR=15.84, FR=4.77, US=4.12, MA=4.12, GM=3.69, IT=3.47, VE=3.25, PE=3.04	Southern Africa=35.36, South America=26.9, Western Europe=6.72, North Africa=6.51, West Africa=5.86, Southern Europe=5.21, North America=4.12, East Africa=2.39
64	■■■■■■■■■■■■■■■■■■■■□□□□■■■■□■■■□□□□■■■■■■■	777777607560771	15	1.21	3.26	LAM6	BR=44.35, US=20.43, GF=5.0, PT=3.04, PE=2.61, FR=2.61, AR=2.39	South America=58.26, North America=20.43, Western Europe=5.22, Southern Europe=3.91, Western Asia=2.61
65	■■■■■■■■■■■■■■■■■■■■■■■■■■■■■■■■□□□□■□□■■■■	777777777760471	60	4.84	42.86	T1	BR=39.29, HT=21.43, US=9.29, FR=6.43, ES=3.57, AT=2.86, CM=2.14	South America=40.71, Caribbean=23.57, Western Europe=12.14, North America=9.29, Southern Europe=4.29, Central Africa=2.14, Northern Europe=2.14
73	■■■■■■■■■■■■□■■■■■■■■■■■■■■■■■■■□□□□■■■□■■■	777737777760731	13	1.05	4.11	T	BR=18.35, IT=11.71, US=11.39, ZA=8.86, FR=6.33, AR=4.43, CN=3.8, MZ=3.16, MX=2.53, GF=2.22	South America=26.9, Southern Europe=13.92, Western Europe=11.39, North America=11.39, Southern Africa=8.86, East Africa=6.33, Eastern Asia=3.8, Central America=3.16, West Africa=2.22, Western Asia=2.22
93	■■■■■■■■■■■■□■■■■■■■□□□□■■■■■■■■□□□□■■■■■■■	777737607760771	28	2.26	5.87	LAM5	VE=19.71, US=15.72, BR=14.47, PE=13.42, HT=10.06, IT=4.4, GP=3.98, ES=2.31, FR=2.31	South America=53.88, Caribbean=15.72, North America=15.72, Southern Europe=6.71, Western Europe=3.77
106	■■■■■■■■□□□■■■■■■■■□□□□□□□□□□□□□□□□□□□■■■■■	776177400000171	20	1.61	13.7	Unknown	ES=32.88, US=15.75, PA=13.01, BR=9.59, CO=4.79, AR=4.79, PE=3.42, FR=3.42, MA=2.74, DZ=2.05	Southern Europe=34.25, South America=23.97, North America=15.75, Central America=13.01, North Africa=4.79, Western Europe=4.79
137	■■■■■■■■■■■■■■■■■□■■■■■■■■■■■■■■□□□□■■□□□□■	777776777760601	12	0.97	1.12	X2	US=68.28, BR=7.46, ZA=6.06, GB=5.13	North America=69.31, South America=8.21, Northern Europe=6.81, Southern Africa=6.06, Western Europe=2.61
244	■■■■■■■■■■■■■■■■■■■■■■■■■■■■■■■■□□□□■■□□□□■	777777777760601	15	1.21	10.64	T1	BR=23.4, ZA=13.48, PT=11.35, BD=9.93, FR=9.22, ZM=5.67, GW=4.96, TZ=4.26, US=3.55, MZ=2.13	South America=24.82, East Africa=14.18, Southern Africa=13.48, Southern Europe=12.77, Southern Asia=11.35, Western Europe=10.64, West Africa=5.67, North America=3.55
863	□□□□□□□□□□□□□□□□□□□□□□□□■■■■□□□□□□□□□□□□□□□	000000007400000	28	2.26	560.0	PINI2	BR=80.0, US=20.0	South America=80.0, North America=20.0
1106	■■■■■■■■■■■■■■■■■■■■□□□□■■■■■■■■□□□□■□□□□□□	777777607760400	38	3.07	172.73	LAM4	PT=40.91, BR=31.82, ES=18.18, IT=4.55, PE=4.55	Southern Europe=63.64, South America=36.36
2512	■■□■□■■■■■■■■□■■■■■■□□□□■■■■■■■■□□□□■■■■■■■	657757607760771	19	1.53	316.67	LAM1	BR=83.33, CL=16.67	South America=100.0

* The 2 letter country codes are according to http://en.wikipedia.org/wiki/ISO_3166-1_alpha-2; countrywide distribution is only shown for SITs with ≥2% of a given SIT as compared to their total number in the SITVIT database.

** Worldwide distribution is reported for regions with more than 2% of a given SIT as compared to their total number in the SITVIT database.
