# Supplementary material for: Clonal expansion across the seas as seen through CPLP-TB database: A joint effort in cataloguing Mycobacterium tuberculosis genetic diversity in Portuguese-speaking countries
Source: Infect Genet Evol. 2019 Aug;72:44–58. doi: 10.1016/j.meegid.2018.03.011 (PMC6598853; doi:10.1016/j.meegid.2018.03.011)
Supplement: Supplementary file 7 — Supplementary Table S3 [file mmc7.pdf]

Supplementary Table S3 – Fifteen-loci MIRU-VNTR clusters found, main associated SITs and distribution by country of origin.

MIRU-VNTR Cluster	Associated SITs	No. of Isolates					
		Angola	Brazil	Guinea-Bissau	Mozambique	Portugal	Total
AO-01	20	2	0	0	0	0	2
AO-02	1548	2	0	0	0	0	2
AO-03	244	2	0	0	0	0	2
AO-04	20	2	0	0	0	0	2
CPLP-01	20, 1755, 42	8	6	0	0	2	16
CPLP-02	20, 2271, 2572	1	0	0	0	2	3
CPLP-03	237	0	1	0	0	1	2
CPLP-04	20	1	1	0	0	0	2
CPLP-05	1530	1	3	0	0	0	4
CPLP-06	1530, 4152	0	1	0	0	1	2
CPLP-07	194, 17	1	2	0	0	1	4
CPLP-08	33, 1224	0	1	0	0	1	2
CPLP-09	nd	1	1	0	0	0	2
CPLP-10	53	1	0	0	0	1	2
GW-01	1	0	0	2	0	0	2
GW-02	1	0	0	3	0	0	3
Lisboa3-A	20, 42	0	0	0	0	11	11
Lisboa3-B	20, 42	0	0	0	0	38	38
POA-01	863	0	4	0	0	0	4
POA-02	17	0	2	0	0	0	2
PT-01	92	0	0	0	0	2	2
PT-02	1	0	0	0	0	3	3
PT-03	1752	0	0	0	0	4	4
PT-04	211	0	0	0	0	2	2
PT-05	53	0	0	0	0	2	2
PT-06	1	0	0	0	0	2	2
PT-07	1106	0	0	0	0	4	4
PT-08	20, 2271	0	0	0	0	3	3
PT-09	20, 42	0	0	0	0	2	2
Q1	290, 42, 1106	1	0	0	0	26	27
RG-01	2512, 729	0	20	0	0	0	20
RG-02	17	0	2	0	0	0	2
RG-03	65, 45, 2512, 4145	0	27	0	0	0	27
RG-04	nd	0	2	0	0	0	2
RG-05	45	0	13	0	0	0	13
RG-06	nd	0	2	0	0	0	2
RG-07	20	0	2	0	0	0	2
RG-08	45	0	2	0	0	0	2
RG-09	20	0	8	0	0	0	8
RG-10	65	0	4	0	0	0	4
RG-11	65	0	2	0	0	0	2

RG-12	nd	0	2	0	0	0	2
RG-13	nd	0	2	0	0	0	2
RG-14	602	0	2	0	0	0	2
RG-15	45	0	2	0	0	0	2
RG-16	45	0	2	0	0	0	2
RG-17	137	0	2	0	0	0	2
RG-18	53	0	10	0	0	0	10
RG-19	602	0	2	0	0	0	2
RG-20	nd	0	2	0	0	0	2
RG-21	45	0	3	0	0	0	3
RG-22	33	0	2	0	0	0	2
RG-23	53, 50	0	2	0	0	0	2
RG-24	60, 177	0	2	0	0	0	2
RG-25	20, 65	0	5	0	0	0	5
RG-26	177	0	2	0	0	0	2
RG-27	33	0	4	0	0	0	4
RG-28	60	0	2	0	0	0	2
RG-29	65	0	4	0	0	0	4
RG-30	65	0	2	0	0	0	2
RG-31	42, 4157	0	4	0	0	0	4
RG-32	nd	0	2	0	0	0	2
RG-33	nd	0	2	0	0	0	2
RG-34	4148	0	2	0	0	0	2
RG-35	nd	0	2	0	0	0	2
RS-01	58	0	2	0	0	0	2
RS-02	50	0	2	0	0	0	2
RS-03	65	0	24	0	0	0	24
RS-04	106	0	4	0	0	0	4
RS-05	863, 4157	0	4	0	0	0	4
RS-06	99	0	2	0	0	0	2
RS-07	53	0	2	0	0	0	2
RS-08	53	0	3	0	0	0	3
RS-09	179	0	2	0	0	0	2
RS-10	453	0	2	0	0	0	2

nd - not determined
