# Supplementary material for: Clonal expansion across the seas as seen through CPLP-TB database: A joint effort in cataloguing Mycobacterium tuberculosis genetic diversity in Portuguese-speaking countries
Source: Infect Genet Evol. 2019 Aug;72:44–58. doi: 10.1016/j.meegid.2018.03.011 (PMC6598853; doi:10.1016/j.meegid.2018.03.011)
Supplement: Supplementary file 8 — Supplementary Table S4 [file mmc8.pdf]

Supplementary Table S4 – Twenty-four-loci MIRU-VNTR clusters found, main associated SITs and distribution by country of origin.

MIRU-VNTR Cluster	Associated SITs	No. of Isolates					
		Angola	Brazil	Guinea-Bissau	Mozambique	Portugal	Total
AO-01	20	2	0	0	0	0	2
AO-02	1548	2	0	0	0	0	2
AO-03	244	2	0	0	0	0	2
AO-04	20	2	0	0	0	0	2
CPLP-01	20, 42	6	1	0	0	1	8
CPLP-01B	20	1	0	0	0	1	2
CPLP-02	20, 2271,2572	1	0	0	0	2	3
CPLP-03	237	0	1	0	0	1	2
CPLP-07	17, 194	1	0	0	0	1	2
CPLP_11	42, 290	1	0	0	0	1	2
GW-01	1	0	0	2	0	0	2
GW-02	1	0	0	3	0	0	3
Lisboa3-A	20, 42	0	0	0	0	9	9
Lisboa3-B	20, 42	0	0	0	0	37	37
POA-01	863	0	3	0	0	0	3
POA-02	17	0	2	0	0	0	2
PT-01	92	0	0	0	0	2	2
PT-02	1	0	0	0	0	3	3
PT-03	1752	0	0	0	0	4	4
PT-04	211	0	0	0	0	2	2
PT-05	53	0	0	0	0	2	2
PT-06	1	0	0	0	0	2	2
PT-07	1106	0	0	0	0	4	4
Q1	1106	0	0	0	0	22	22
RS-01	58	0	2	0	0	0	2
RS-02	50	0	2	0	0	0	2
RS-03	65	0	8	0	0	0	8
RS-04	106	0	4	0	0	0	4
RS-05	863	0	3	0	0	0	3
RS-06	99	0	2	0	0	0	2
RS-08	53	0	3	0	0	0	3
RS-09	179	0	2	0	0	0	2
RS-10	453	0	2	0	0	0	2
